# Local Clonal Diversification and Dissemination of B Lymphocytes in the Human Bronchial Mucosa

**DOI:** 10.3389/fimmu.2018.01976

**Published:** 2018-09-07

**Authors:** Line Ohm-Laursen, Hailong Meng, Jessica Chen, Julian Q. Zhou, Chris J. Corrigan, Hannah J. Gould, Steven H. Kleinstein

**Affiliations:** ^1^Randall Centre for Cell and Molecular Biophysics and School of Basic and Medical Biosciences, King's College London, London, United Kingdom; ^2^Medical Research Council and Asthma UK Centre in Allergic Mechanisms of Asthma, London, United Kingdom; ^3^Department of Pathology, Yale School of Medicine, New Haven, CT, United States; ^4^Interdepartmental Program in Computational Biology and Bioinformatics, Yale University, New Haven, CT, United States; ^5^Department of Respiratory Medicine and Allergy and School of Immunology and Microbial Sciences, King's College London, London, United Kingdom; ^6^Department of Immunobiology, Yale School of Medicine, New Haven, CT, United States

**Keywords:** asthma, B-cell antibody repertoire, bronchial mucosa, clonal dissemination, cell trafficking, next generation sequencing, somatic hypermutation

## Abstract

The efficacy of the adaptive humoral immune response likely requires diverse, yet focused regional B cell antibody production throughout the body. Here we address, in the first study of its kind, the B cell repertoire in the bronchial mucosa, an important barrier to antigens inhaled from the atmosphere. To accomplish this, we have applied high-throughput Adaptive Immune Receptor Repertoire Sequencing (AIRR-Seq) to 10 bronchial biopsies from altogether four different sites in the right lungs from an asthmatic patient and a healthy subject. While the majority of identified B cell clones were restricted to a single site, many were disseminated in multiple sites. Members of a clone were shared more between adjacent biopsies than between distal biopsies, suggesting local mucosal migration and/or a homing mechanism for B cells through the blood or lymph. A smaller fraction of clones spanned the bronchial mucosa and peripheral blood, suggesting ongoing trafficking between these compartments. The bronchial mucosal B cell repertoire in the asthmatic patient was geographically more variable but less diverse compared to that of the healthy subject, suggesting an ongoing, antigen-driven humoral immune response in atopic asthma. Whether this is a feature of atopy or disease status remains to be clarified in future studies. We observed a subset of highly mutated and antigen-selected IgD-only cells in the bronchial mucosa. These cells were found in relative high abundance in the asthmatic individual but also, albeit at lower abundance, in the healthy subject. This novel finding merits further exploration using a larger cohort of subjects.

## Introduction

B cells form an essential plank of the adaptive immune response to environmental assaults at mucosal surfaces. The airways mucosa is a very large surface open to such assault, with B cells situated in the bronchial mucosa between the pseudostratifed squamous epithelium of the airways on the outside and the mucosal parenchyma containing blood and lymphatic capillaries and flanked by smooth muscle and cartilage on the inside (1).

We have previously shown that B cells in the bronchial mucosa are activated in response to environmental assaults in an antigen-specific fashion and undergo somatic hypermutation (SHM) with class switching, affinity maturation and plasma cell differentiation to produce high-affinity antibodies at the site of the assault (2, 3). Nevertheless, because of the practical difficulties associated with multiple and repetitive sampling of the mucosa in humans, there currently exists remarkably little direct evidence as to whether proliferating and maturing B cells remain confined locally within the mucosa near the site of activation, or alternatively whether they become widespread geographically at an early stage, and how this process is influenced by ongoing mucosal inflammation.

Recent advances in technology, particularly with high-throughput Adaptive Immune Receptor Repertoire Sequencing (AIRR-Seq) have provided scientists with a unique new tool with which to pinpoint sites of B cell maturation and uncover trafficking pathways within and between organs (4). We have previously used this technology to show evidence of allergen-driven SHM in the nasal mucosa of hay fever sufferers during the pollen season (5). We have also used it to uncover B cell trafficking between the central nervous system and lymphoid tissues in patients with multiple sclerosis (6). Others have observed distinct antibody repertoires in mucosal tissue compared to blood and lymphoid tissue in healthy individuals (7), and a large degree of sharing of sequences (“connectivity”) between different regions of the gastrointestinal tract (8, 9). Within the gastrointestinal tract the immunoglobulin repertoires from various sites were found to be highly related to each other but to be less related to the antibodies from the blood, bone marrow, spleen and lung (9).

In the present study we have devised a more extensive strategy to study the maturation of B cells *in situ* within the bronchial mucosa in the context of environmentally-induced inflammation, using asthma as an archetypal example of this phenomenon. Our strategy was to obtain two or three bronchial biopsies from each of four specific sites within the bronchial tree extending from the carina to the third or fourth generation of the bronchial tree from one asthmatic (*AA*) and one healthy subject (*NANA*) and to use AIRR-Seq to examine in great detail the diversity of the immunoglobulin receptor repertoire and the interrelationships between this repertoire in distinct areas of the bronchial mucosa as well as the peripheral blood.

Our experiments were designed to determine (1) whether or not the bronchial mucosa is a site of activation of B cells as evidenced by *in situ* SHM and immunoglobulin class switching; (2) whether or not the bronchial mucosal immunoglobulin repertoire is diverse or restricted in terms of isotypes and gene usage and shows signs of antigen-driven selection; and (3) whether or not locally clonally expanded cells are able to migrate to more remote sites within the bronchial mucosa and the peripheral blood.

## Materials and methods

### Participants

Bronchial biopsies and peripheral blood were obtained from one atopic asthmatic (*AA*) and one non-atopic non-asthmatic (*NANA*). The study was conducted at and in accordance with the recommendations of King's College London and Guy's and St Thomas' NHS Foundation Trust and the protocol was approved by the London Central Research Ethics Committee (REC number 15/LO/1800). Both subjects gave written informed consent in accordance with the Declaration of Helsinki. The criteria for the diagnosis of asthma and atopy have been described previously (10).

### Peripheral blood and tissue sample collection and processing

Bronchial biopsies were obtained at fibreoptic bronchoscopy from four distinct sites within the airways of the right lung, namely around the origin of the right main bronchus near the carina and then around the origins of 1st, 2nd, and 3rd or 1st, 3rd, and 4th generation bronchi further down the bronchial tree as shown later. Samples were obtained in reverse order (from the base of the lung upwards) to minimize cross-contamination. A total of 3-5 biopsies was obtained from each site, equating to a total of 15 from the atopic asthmatic *AA* and 12 from the healthy subject *NANA*, although only 10 biopsies were analyzed from each individual. Biopsies were immediately immersed in RNAlater (Life Technologies) and stored at 4°C overnight prior to long term storage in liquid nitrogen. Two or three biopsies from each site were chosen for sequencing based on the highest yields of RNA. 40 ml of venous blood were collected into 1:10 ACD (anticoagulant-citrate-dextrose) (Sigma-Aldrich) immediately before the bronchoscopy procedure. PBMCs were isolated by centrifugation over Ficoll-Paque (GE Health Care) according to the manufacturer's instructions and stored in freezing medium (10% dimethyl sulphoxide in fetal calf serum) in liquid nitrogen. Aliquots of 10^7^ cells were used in the following RNA extraction step.

### RNA extraction and next generation sequencing

Total RNA was extracted from the biopsies by disrupting the tissue in a TissueLyser (Qiagen) using 5 mm stainless steel beads, followed by centrifugation through a Qiashredder (Qiagen) and extraction using the RNeasy Plus Micro Kit (Qiagen), according to manufacturers' instructions. PBMC RNA was extracted using the RNeasy Plus Mini Kit (Qiagen). Both kits included a step to remove genomic DNA. All the RNA extracted from each biopsy and a minimum of 1 mg PBMC RNA was shipped to iRepertoire (iRepertoire Inc., Huntsville, Alabama, USA) for sequencing of the immunoglobulin heavy chain repertoire on the Illumina MiSeq platform with 250 bp paired-end reads. iRepertoire use their own patent protected primers including VH-gene family specific forward primers in the heavy chain FWR1 region and isotype-specific primers in the CH1 constant region. All samples were sequenced in two sequencing runs.

### Sequencing data processing

iRepertoire provided filtered and merged reads processed as previously described (11). Additional data processing is described in detail in the Supplementary Methods section. Briefly, the sequences from iRepertoire were analyzed with ImMunoGeneTics (IMGT)/High V-QUEST to determine V(D)J assignments (12). The data were then processed with the Change-O v0.3.2 (13), Alakazam v0.2.5 (13) SHazaM v0.1.4 (13), and other custom scripts through the R environment (14). Filtering steps included discarding sequences with mismatches in the constant region, sequences with more than six mismatches in a 10 nucleotide stretch and sequences with a copy number below five (below one for IgE). Non-functional sequences were also removed from the data, and clonally related sequences were identified using a clustering-based approach as detailed in the Supplementary Methods (13). A detailed description of the various analyses performed can be found in figure legends and Supplementary Methods. In accordance with AIRR Community recommendations (15) the data set can be found at NCBI's sequence read archive (SRA), https://www.ncbi.nlm.nih.gov/sra, under BioProject accession PRJNA422604.

## Results

### B cells in the human bronchial mucosa have a highly diverse immunoglobulin repertoire

Ten bronchial biopsies from four distinct sites in the bronchial mucosa were collected at fibreoptic bronchoscopy from two individuals, one atopic asthmatic (*AA*) and one non-atopic, non-asthmatic healthy subject (*NANA*). A peripheral blood sample was also collected at the same time. Total RNA was extracted from the tissue and blood samples and the immunoglobulin heavy chains were amplified from the end of framework region 1, FWR1, and into the 5′ end of the heavy chain CH1 domain and subsequently sequenced using Adaptive Immune Receptor Repertoire Sequencing (AIRR-Seq) on the Illumina MiSeq platform. A total of 6,782,411 sequences (*AA*: 3,129,960 and *NANA*: 3,652,451) were generated. After quality control (QC), filtering and trimming, a total of 238,928 unique sequences (*AA*: 121,507, and *NANA*: 117,421) of high quality were retained and used for the analyses described below (Table S1).

**Figure 1:**
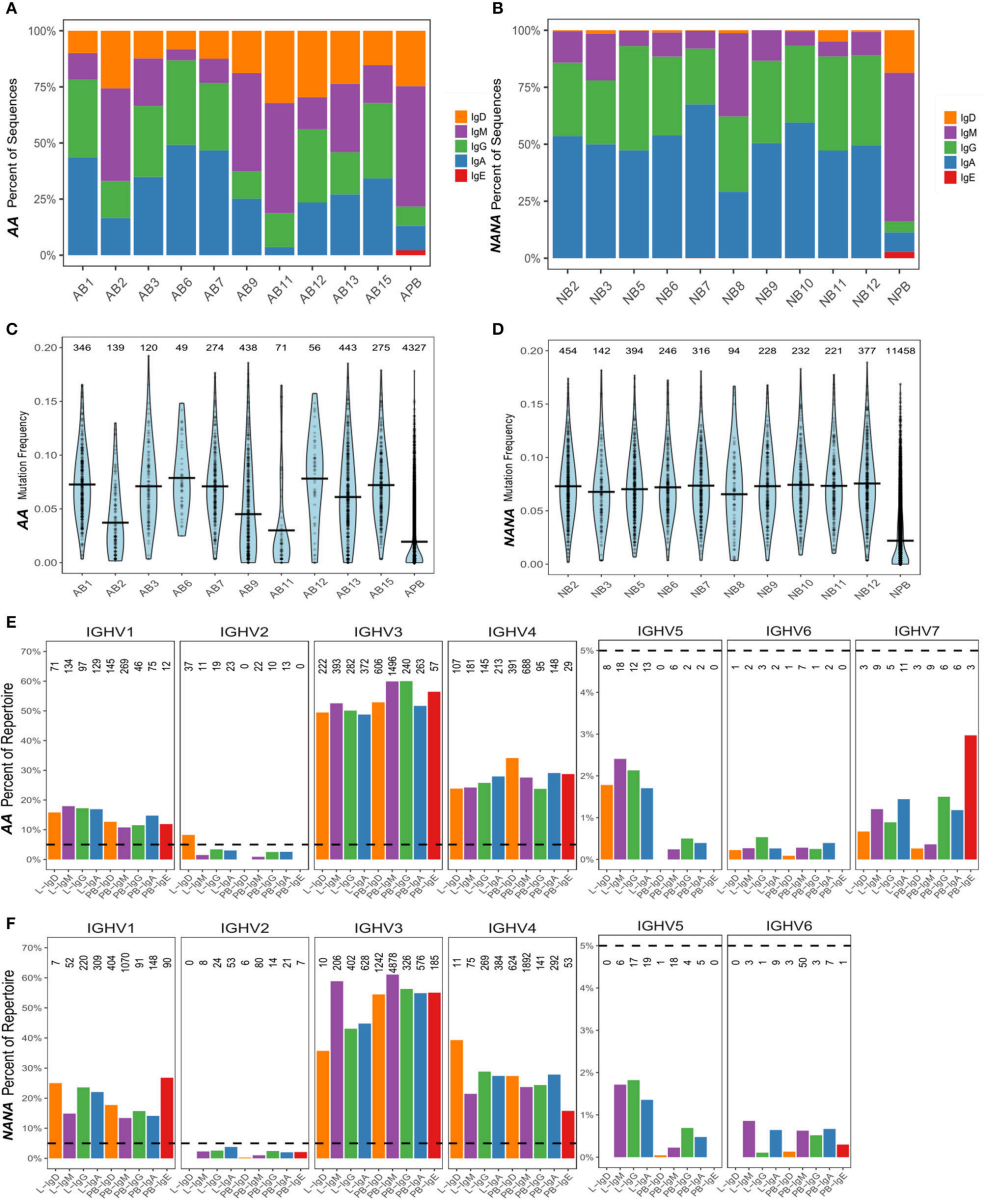
The human bronchial mucosal repertoire is highly diverse and distinct from the peripheral blood repertoire. For each biopsy and peripheral blood sample from **(A)** the asthmatic patient *AA* and **(B)** the healthy subject *NANA*, the percentages of IgD (orange), IgM (purple), IgG (green), IgA (blue), and IgE (red) clones were calculated as a fraction of the total numbers of clones [the numbers of clones in each sample are the same as in **(C,D)**]. The mutation frequencies for each clone were calculated as described in the Supplementary Methods, and the distribution of the frequencies in each sample (10 individual biopsies and one peripheral blood sample) determined as shown in **(C)** *AA* and **(D)** *NANA*. Horizontal lines show the mean values and each point represents a clone, with the numbers of clones in each sample stated above the violins. The VH-gene segment usages by clones of different isotypes from all of the bronchial mucosal samples (all 10 biopsies combined) and the peripheral blood were calculated as the percentages of clones assigned to a given VH-gene family within the total number of clones for each isotype by Alakazam v0.2.5 (13) and plotted for **(E)** *AA* and **(F)** *NANA*. A dotted line is shown at 5% to highlight the different scaling of the y-axis for VH1-VH4 and VH5-VH7. No sequences using VH7 were found in any of the samples from *NANA*. The numbers of clones in each category are shown above each bar.

We found that all of the bronchial mucosal biopsies from *AA* contained a mixed repertoire of IgD, IgM, IgG and IgA clones (Table 1 and Figure 1A). No IgE clones were found (see Discussion). The pattern was distinct from that in the biopsies from *NANA* where fewer IgM and practically no IgD clones were identified (Table 1 and Figure 1B), compatible with the hypothesis that, in healthy individuals, principally mature, isotype switched memory B cells reside in the bronchial mucosa. This is further supported by the finding that the mean mutation frequency of the clones from *NANA* was relatively constant (~7%) in all 10 biopsies (Figure 1D), whereas the mean mutation frequency varied from ~4 to 8% in individual biopsies from *AA* (Figure 1C) with biopsies featuring the highest percentages of IgM clones (AB2, AB9, and AB11, see Figure 1A) showing the lowest mean mutation frequency. For all isotypes, the clones from *AA* contained a wider range in terms of numbers of sequences per clone than those from *NANA* (Table 1). Together with the finding of high proportions of IgD and IgM clones in some of the biopsies from *AA*, these findings are compatible with the hypothesis that the inflamed bronchial mucosa is a site of local recruitment of naïve B cells.

**Table 1:** Numbers of unique sequences and clones retained after all filtering and quality control steps.

	***AA*** **Bronchial mucosa^a^**		***AA*** **Blood**		***NANA*** **Bronchial mucosa^a^**		***NANA*** **Blood**	
	**No of sequences**	**No of clones**	**No of sequences**	**No of clones**	**No of sequences**	**No of clones**	**No of sequences**	**No of clones**
**IgD**	7,424	449 (1–1,281; 3)^*^	1,252	1,146 (1–10; 1)^*^	411	28 (1–304; 3)^*^	2,670	2,280 (1–25; 1)^*^
**IgM**	19,346	748 (1–7,718; 3)^*^	3,227	2,497 (1–42; 1)^*^	2,911	350 (1–416; 3)^*^	10,635	7,988 (1–33; 1)^*^
**IgG**	62,867	563 (1–6,808; 11)^*^	5,585	400 (1–1,388; 5)^*^	71,728	933 (1–2,178; 20)^*^	4,061	579 (1–79; 4)^*^
**IgA**	20,126	763 (1–5,238; 3)^*^	1,458	509 (1–74; 2)^*^	19,616	1,402 (1–1,906; 4)^*^	2,412	1,049 (1–25; 1)^*^
**IgE**	0	0	222	101 (1–83; 1)^*^	1	1 (1–1; 1)^*^	2,976	336 (1–1,387; 1)^*^
**Total**	109,763	1,719 (1–7,718; 4)^*^	11,744	4,327 (1–1,388; 1)^*^	94,667	2,090 (1–2,178; 6)^*^	22,754	11,458 (1–1,387; 1)^*^

*AA: atopic asthmatic patient, NANA: non-atopic, non-asthmatic individual*.

a *All ten biopsies from the individual combined*.

* *The numbers in the parenthesis give the range (minimum to maximum) and the median of the number of sequences per clone*.

**Figure 2:**
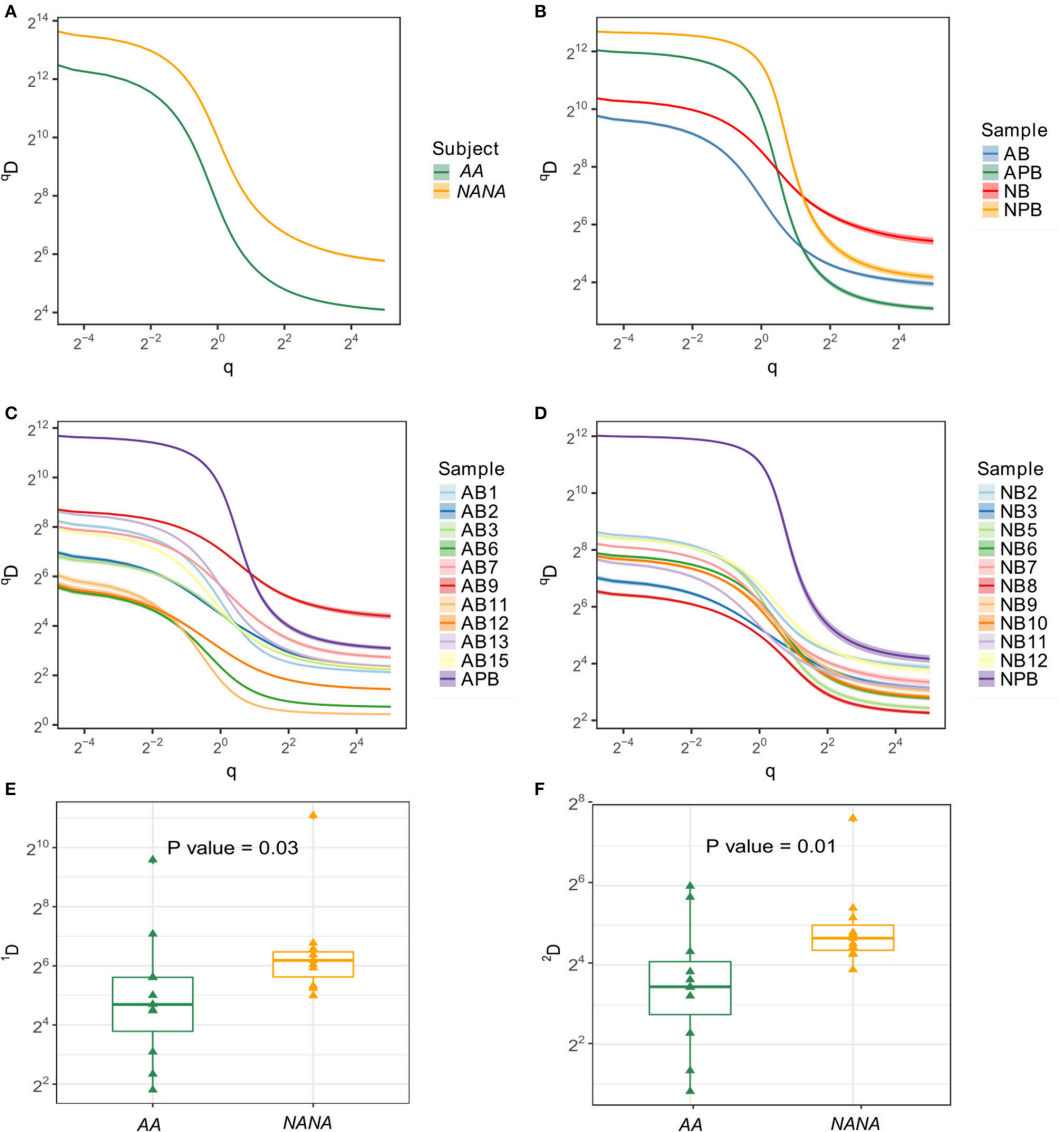
Samples from the asthmatic subject *AA* show less diversity than those from the healthy individual *NANA*. Clonal diversity analysis was performed by Alakazam v0.2.5 (6, 13) using the generalized diversity index proposed by Hill (16) with uniform resampling to correct for sequencing depth. The diversity index (^q^D) was calculated over a range of diversity orders (q) and plotted as a smooth curve for **(A)** all sequences sampled from *AA* or *NANA***, (B)** all biopsy samples combined (AB and NB) with peripheral blood as a separate entity (APB and NPB) from *AA* (prefix; A) and *NANA* (prefix; N), respectively**, (C)** all individual samples from *AA* and **(D)** all individual samples from *NANA*. The Shannon diversity index **(E)**, which equates to *q* = 1, and the Simpson diversity index **(F)**, *q* = 2, were plotted for all individual biopsies from *AA* and *NANA*. Horizontal lines indicate the median and the box the 25–75th percentiles.

Ongoing immune activity is often characterized by clonal expansion of B cells and an accompanying loss of clonal diversity (9). To compare this diversity further we used the method of Hill (16) to measure multiple aspects of diversity in both subjects. In terms of numbers of distinct clones and size of the most expanded clone, we found that the repertoire of the healthy subject was more diverse than that of the atopic asthmatic. This was true when considering all sequences together and separately in the bronchial mucosa and peripheral blood (Figures 2A,B). With the exception of biopsy 9 from *AA*, the diversity appeared to be greater in peripheral blood than in individual biopsies (Figures 2C,D). It is interesting to note that the Hill plots for individual biopsies from *NANA* were more uniform than those from the *AA*, supporting the conclusion from the mutation analysis that the between-biopsy variability was larger in *AA* compared with *NANA*. In terms of diversity, the mucosal antibody repertoire from *NANA* was significantly more diverse than that from the asthmatic patient *AA* as seen from the Shannon and Simpson indices (*P* = 0.03 and 0.01, respectively) (Figures 2E,F). Overall, the bronchial mucosa of the asthmatic subject contained fewer unique sequences with a greater degree of clonal expansion, suggesting a narrowing of overall diversity consistent with an ongoing immune response.

It has previously been found that genes from the VH5 family are over-expressed in IgE from B cells in the asthmatic bronchial mucosa (17). Prompted by this, we analyzed the VH gene usage of individual clones from B cells from the bronchial mucosa and peripheral blood of our two subjects. The repertoires were dominated by VH3 and VH4 family genes followed by VH1 (Figures 1E,F), which is expected for peripheral blood from previously reported findings (18). Very few of the clones used genes from VH2, VH5, VH6, and VH7 families, with no VH7 genes from any of the samples from *NANA*. Despite the overall low usage, we did find that VH5 was significantly over expressed in the bronchial mucosa compared to the peripheral blood of both subjects (*P* < 0.05, Chi-squared). This was true for all isotypes except for IgD from *NANA* where the number of bronchial mucosal clones identified (28 in total) was insufficient for this type of analysis. There were no striking differences in the patterns of VH gene usage between *AA* and *NANA*. Thus, the bronchial mucosa immunoglobulin repertoire was highly diverse in terms of VH gene usage, but exhibited a significant bias toward VH5 family usage in the bronchial mucosa compared to the peripheral blood.

### Mutation patterns are consistent with antigen-driven selection

**Figure 3:**
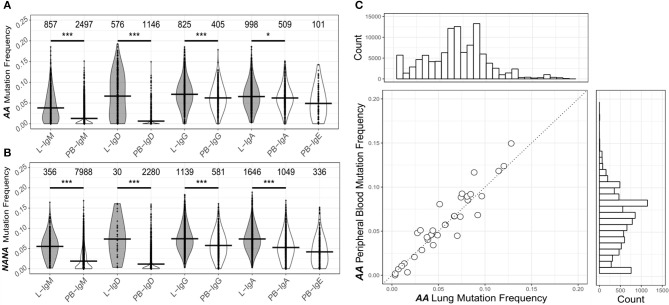
The human bronchial mucosa repertoire is diversified by SHM and connected to the peripheral blood compartment. The clones were grouped by isotype and the mutation frequency for each clone determined separately in the bronchial mucosa (10 biopsies combined, gray violins) and peripheral blood (white violins) of **(A)** the asthmatic patient *AA* and **(B)** the healthy subject *NANA* (see Supplementary Methods). No IgE sequences were found in the bronchial mucosa samples. The lines indicate the median mutation frequencies, while the numbers above the violins indicate the numbers of clones analyzed. *P < 0.05 and ***P < 0.001 indicate that the median mutation frequencies in the bronchial mucosa and peripheral blood samples were statistically significantly different for all comparisons in both individuals. **(C)** For each clone (circle) from *AA* that contained sequences from both bronchial mucosa and peripheral blood, the median mutation frequencies were calculated separately for sequences from the bronchial mucosa and the peripheral blood and compared, the dashed line showing equivalence. The histogram above the plot shows the mutation frequency distribution for all unique sequences from the bronchial mucosa and the histogram on the right the distribution for all unique sequences from the peripheral blood.

To investigate the levels of SHM in more depth we compared the mutation frequencies found in mucosa-derived clones to those from peripheral blood. For all isotypes in both subjects, with the exception of IgE for which we could detect no bronchial mucosal-derived sequences, we found that clones from the mucosa were significantly more mutated than those from the peripheral blood (Figures 3A,B). Despite these overall differences in the mutation frequencies, sequences from bronchial mucosa and peripheral blood derived from the same clone showed similar mutation frequencies (Figure 2C). The clones with members in both tissue and peripheral blood spanned the entire range of mutation frequencies and included clones in germline configuration (Figure 3C). Overall, the mutation frequencies that we observed in clones from the peripheral blood were consistent with previous findings (19) in both individuals, with IgG and IgA showing the highest mutation frequency (*ca*. 6%), followed by IgE (*ca*. 5%), and IgM and IgD (*ca*. 1%). The IgM and IgD sequences were mostly unmutated and thus presumably originated from naïve B cells, although the analysis did reveal the presence of some mutated memory sequences. There are no previous, isotype-specific, large-scale data from the bronchial mucosa, but in nasal biopsies from patients with rhinitis Wu *et al*. (5) found an IgM mutation frequency of *ca*. 4.5% (comparable to our *ca*. 4.5% in *AA* and *ca*. 5.5% in *NANA*), and a combined IgA and IgG mutation frequency of about 9%, which is somewhat higher than the 6-7% found in our present data.

**Figure 4:**
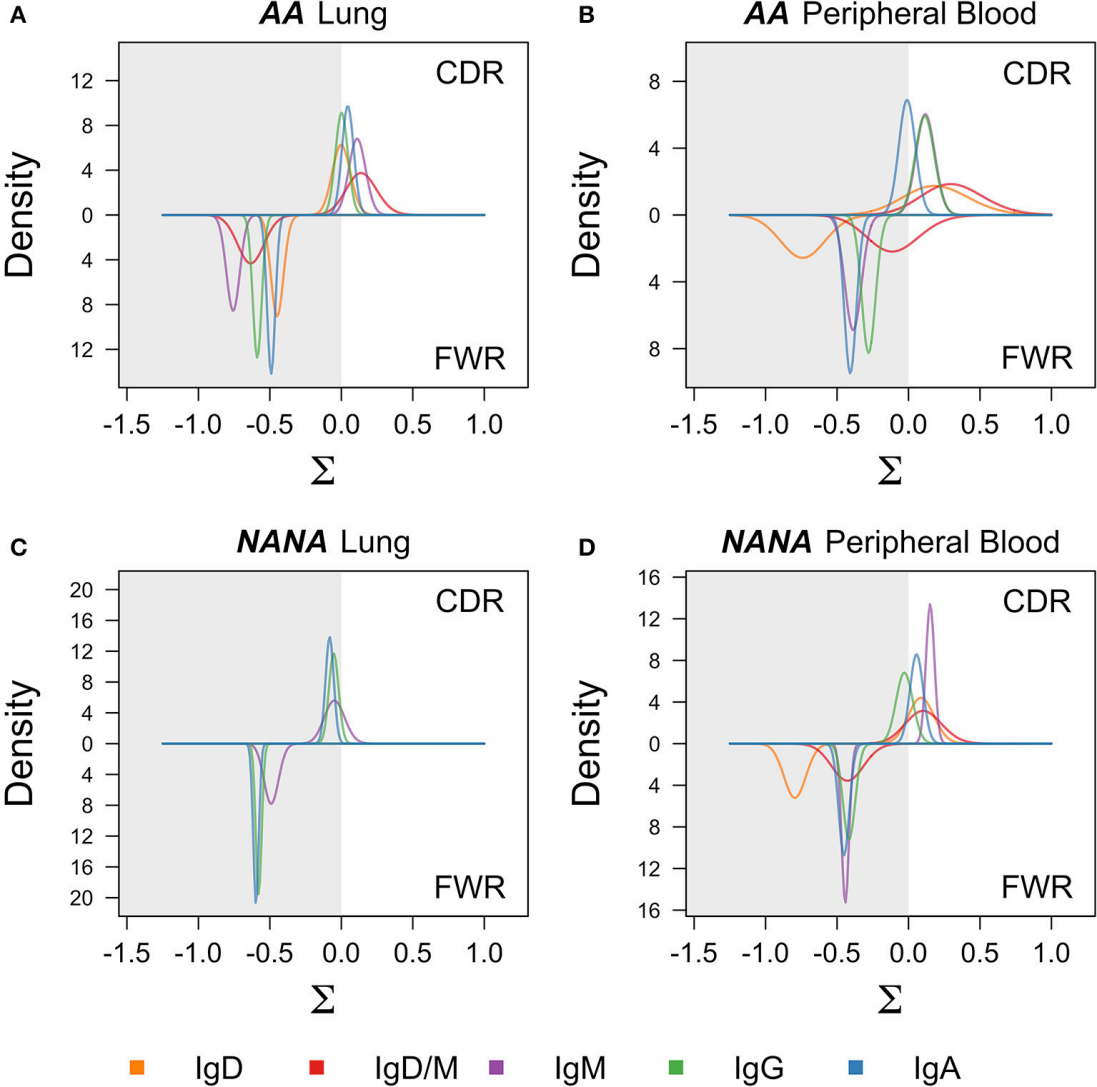
Negative selection in framework regions and neutral to positive selection in complementarity determining regions suggest antigen-driven selection in all cell subsets. BASELINe (24) was used to calculate the posterior distribution of selection strengths (∑) for complementarity determining regions (CDR) and framework regions (FWR) for each isotype in each sample; **(A)** *AA* bronchial mucosa (*AA* lung), **(B)** *AA* peripheral blood, **(C)** *NANA* bronchial mucosa (*NANA* lung), and **(D)** *NANA* peripheral blood were calculated and plotted as smooth curves. IgD and IgD/M for *NANA* bronchial mucosa were excluded because an insufficient number of sequences was available for analysis. Curves for the FWRs are shown in the lower half and curves for the CDRs are shown in the upper half of each panel. The white areas indicate positive selection and the gray areas indicate negative selection. Whereas the selection strength on the FWR is negative in all samples, it is slightly positive on the CDR in *AA* bronchial mucosa, *AA* peripheral blood and *NANA* peripheral blood, but neutral in *NANA* bronchial mucosa. All calculations were done using the “calcBaseline” and “groupBaseline” functions from SHazaM (13) (v0.1.4).

The process of SHM is driven by activation-induced cytidine deaminase (AID) and error-prone repair pathways, both of which have a well-characterized sequence bias (20, 21). In conventional germinal center reactions, the mutation process itself is followed by selection that further shapes the mutation patterns seen in antigen-driven selection (22). To investigate whether these same processes were driving the observed diversification in the bronchial mucosal repertoire, we first analyzed the distribution of mutations across the sequences. As expected, most mutations were located in the complementarity determining regions (CDRs) with spikes in known hotspot areas, including framework region 3 (FWR3) (Figures S1A,B). We refined the analysis to look at the mutability of DNA 5-mer motifs containing known SHM hot-, cold- and neutral spots (Figure S2A). This analysis showed that, across all isotypes and all sample types in both individuals, mutations occurred more frequently in the hotspots than in the cold and neutral spots. These mutation patterns are consistent with typical SHM targeting (21). To test whether the mutation patterns showed evidence of antigen-driven selection, we used the algorithm BASELINe (23) (see Supplementary Methods) to quantify selection strength. The selection pressure was slightly positive in the CDRs of the clones of all isotypes from the peripheral blood from both *AA* and *NANA* (Figures 4B,D), and in the bronchial mucosal clones from *AA* (Figure 4A). It was neutral in the bronchial mucosal clones from *NANA*, suggesting a lesser degree of antigen-driven selection in this compartment (Figure 4C). Although these data also suggest slight variation according to antibody isotype, a more detailed analysis with a larger cohort of patients will be required in future studies to clarify whether and how far the degree of selection is isotype-dependent. The selection pressure was negative in all FWRs consistent with previous results, and the expectation that many amino acid changes in this region will be selected against in order to maintain structural integrity (24). These data are consistent with a scenario of maturation driven by typical SHM along with antigen-driven selection of the B cells in the bronchial mucosa.

### B cell clones are widely dispersed throughout the bronchial mucosa

**Figure 5:**
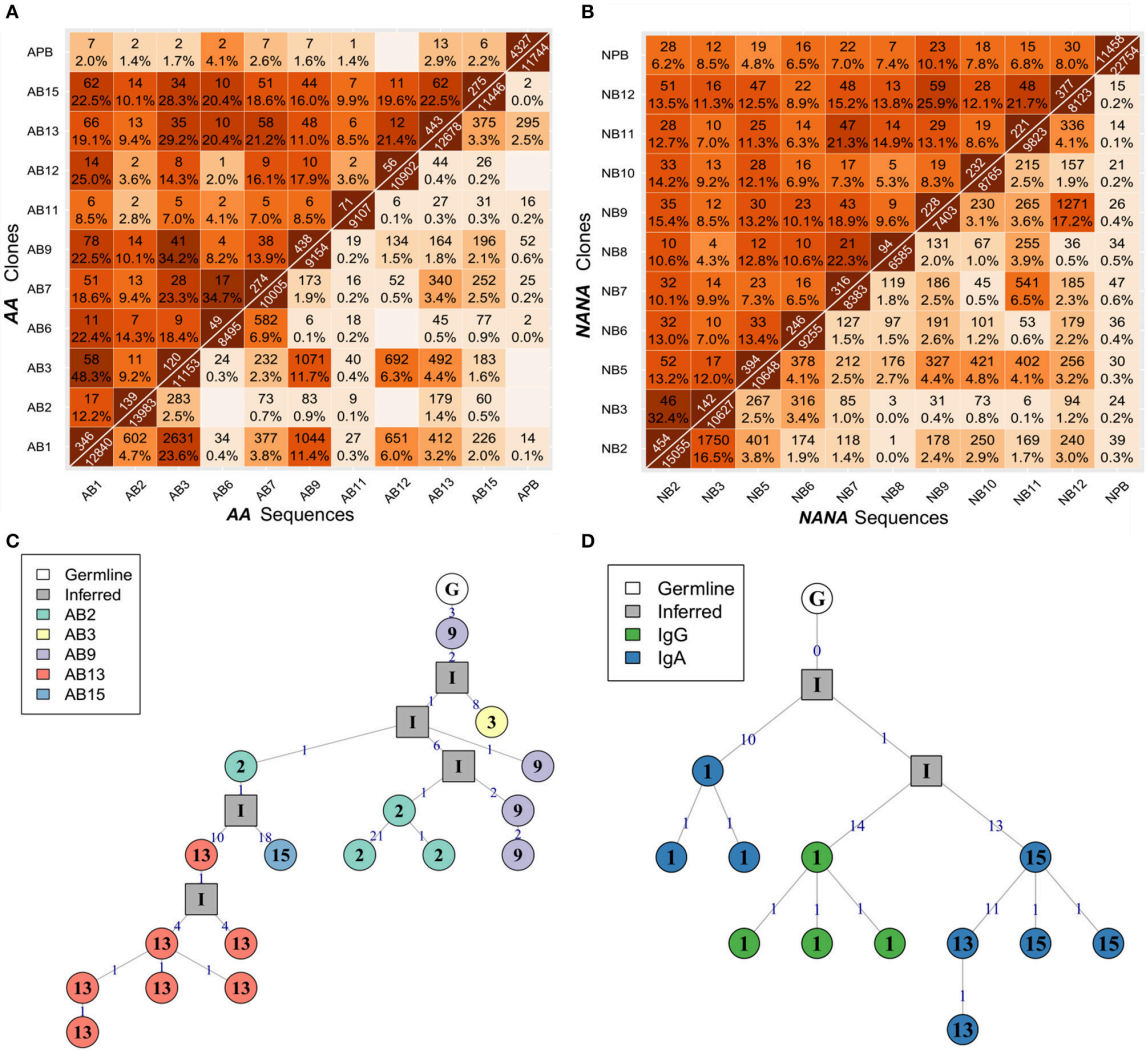
B cells migrate throughout the bronchial mucosa. The numbers of shared clones (top triangle) and sequences (bottom triangle) between all pairs of samples were calculated for **(A)** the asthmatic patient *AA* and **(B)** the healthy subject *NANA*. Numbers on the diagonal indicate the total numbers of clones (top) or unique sequences (bottom) per sample, with the off-diagonal numbers indicating the numbers of shared clones or sequences respectively. Percentages were calculated by dividing by the number of clones or unique sequences in the smaller of the two samples, and darker colors indicate higher overlap. Sequence overlap required nucleotide equivalence across the entire VDJ region, but not the constant region. **(C)** A lineage tree for a heavily expanded IgM clone from *AA* with members distributed across multiple mucosal biopsies. The biopsy sample is indicated by the number within each node, and the number on each line denotes the number of SHM. **(D)** A lineage tree of a dual isotype, expanded clone from *AA* with members from biopsy 1 (IgG and IgA) and biopsies 13 and 15 (IgG only). Square nodes with an “I” label indicate inferred sequences.

To determine whether the clonal expansion observed in the bronchial mucosa B cells was associated with migration throughout the tissue, we analyzed the pattern of sharing across the multiple biopsies within the bronchial mucosa. All possible pairs of biopsies shared several clones (Figures 5A,B - upper triangle), with the degree of overlap ranging from 2% (one of 49 AB6 clones related to AB12) to 48% (58 of 120 AB3 clones related to AB1) of all clones per biopsy pair. In most cases, we were even able to identify identical sequences from biopsy pairs (Figures 5A,B - lower triangle) but the percentages were lower than for clonal overlap (no overlap between AB6 and AB12 to 0.3% (24 of 11153 sequences) between AB3 and AB1), suggesting that the B cells mature and accumulate SHM and/or undergo isotype switching during migration between the biopsy sites. This conclusion was supported by the clonal trees (Figures 5C,D). One example lineage tree (Figure 5C) spanned five biopsies with sequences from each biopsy found in separate branches. A second tree (Figure 5D) displayed a similar pattern of sequences from two different biopsies in separate branches. This tree furthermore showed that two different isotypes could be identified in one of the biopsies, providing an example of an expanded, mutated and isotype-switched clone.

**Figure 6:**
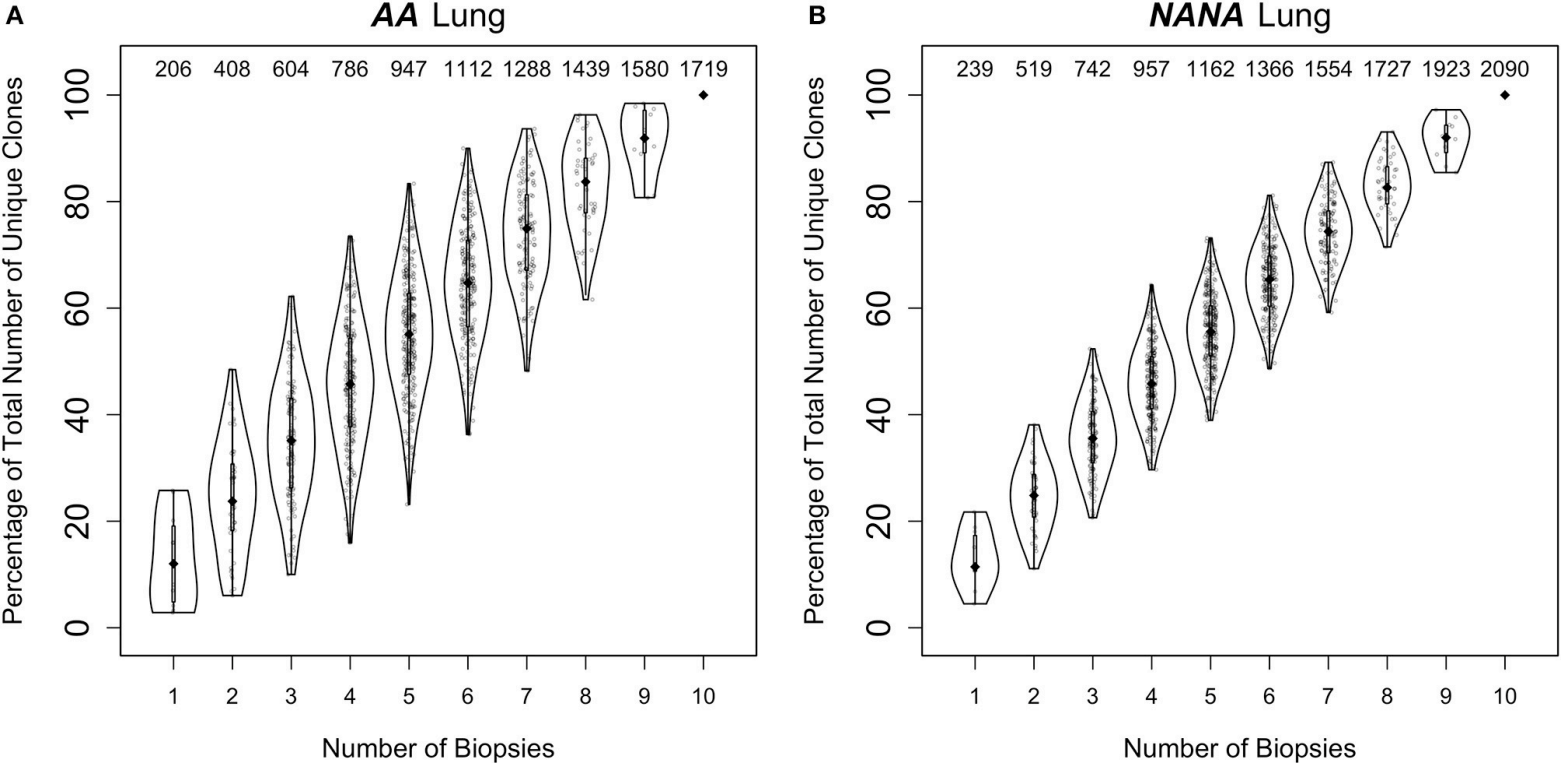
Every biopsy contributes new information about the bronchial mucosal repertoire. The total numbers of clones were determined for all permutations of sampling 1 to 10 biopsies out of a total number of 10 from **(A)** the asthmatic patient *AA* and **(B)** the healthy subject *NANA*. At each subsampling depth of *n* biopsies, there are 10!/[*n*!(10-*n*)!] ways to choose from a total of 10 biopsies. For each of these samplings of *n* biopsies, the percentages of unique clones were calculated relative to the total number of clones when considering all 10 biopsies. The diamond-shaped black dots indicate the median percentages and the gray bars the 25^−^75th quartiles. The numbers at the top indicate the median numbers of unique clones across the permutations.

Despite this high frequency of shared clones within some regions of the bronchial mucosa, the overlap analysis also showed that each biopsy harbored a sizeable number of unique sequences and clones. As multiple biopsies are difficult to obtain, we asked whether each additional biopsy was providing additional information through a clonal diversity rarefaction analysis (Figures 6A,B). This analysis assesses the increase in coverage achieved with each additional biopsy by randomly sampling 1-10 biopsies out of a total number of 10. It can be seen that each additional biopsy contributed more clones to the total repertoire and that the growth in coverage did not reach a plateau in either *AA* or in *NANA*. In conclusion, we found a high degree of clonal sharing between biopsies from different parts of the bronchial mucosa, but we also found that each biopsy contained a high number (range 10–331) of single biopsy origin clones corresponding to 38–76% of all the clones within the biopsy (data not shown).

### Higher connectivity between clones from biopsies taken within close proximity

**Figure 7:**
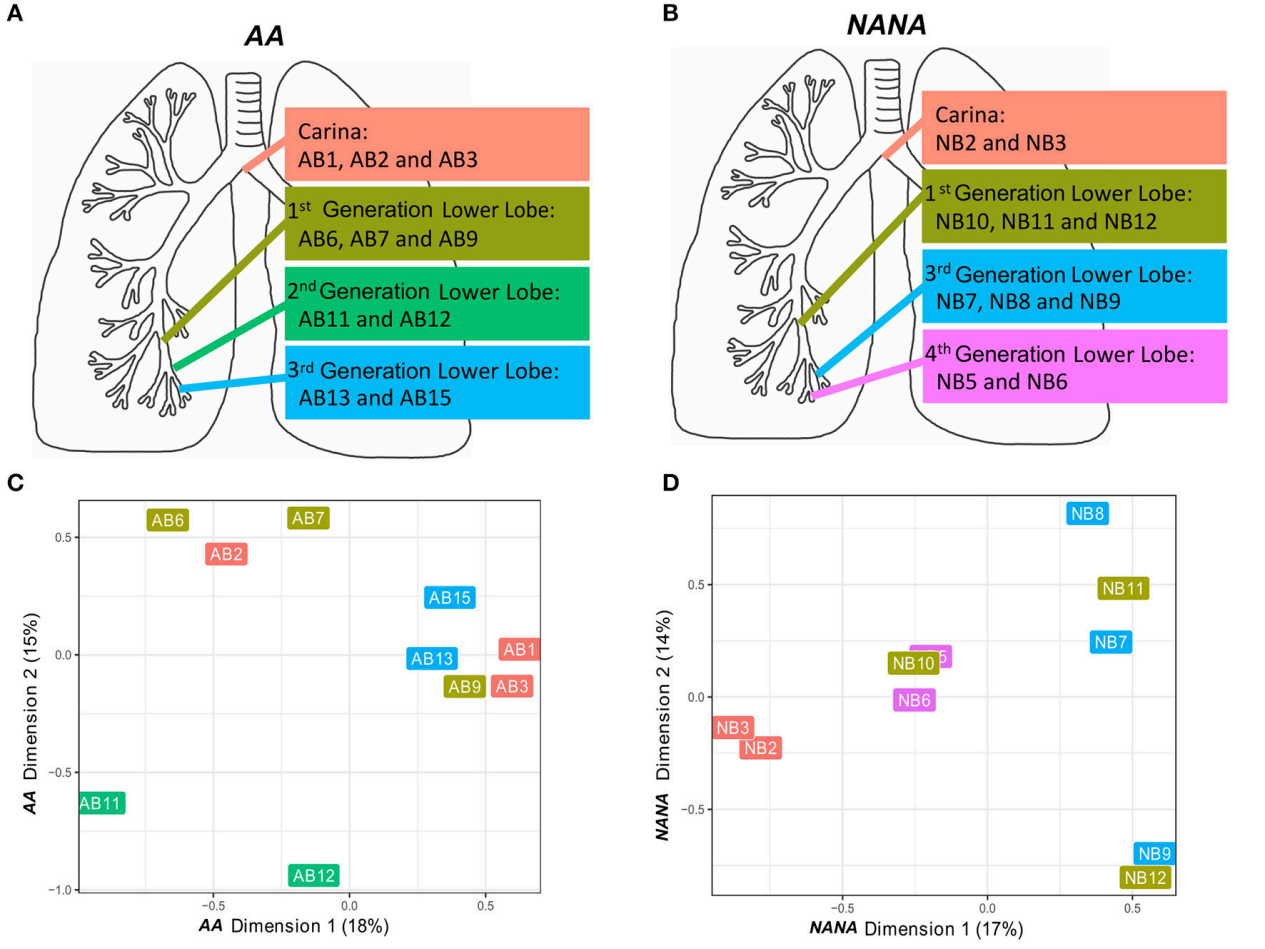
B cells tend to expand locally within the bronchial mucosa. Two or three bronchial biopsies were sampled from each of four distinct sites within the bronchial tree of **(A)** an asthmatic patient *AA* and **(B)** a healthy subject *NANA*. Multidimensional scaling was used to visualize the relatedness of individual samples from **(C)** *AA* and **(D)** *NANA*. Relatedness was defined by the Manhattan distances of normalized clone counts as detailed in Supplementary Methods: “Connectivity analysis.” Label colors correspond to the four distinct sites within the lung in **(A)** *AA* and **(B)** *NANA*.

Based on the high frequency of shared B cell clones within the bronchial mucosa (Figures 5A,B) we wished to investigate whether or not clonal expansion in the bronchial mucosa is focused locally. We calculated the clonal relatedness between all possible sample pairs and used multidimensional scaling to visualize these distances between samples (see Supplementary Methods). Visually it appeared that sequences from biopsies sampled from the same sites in the bronchial tree (Figures 7A,B) tended to be more closely related than those from biopsies from more distal sites (Figures 7C,D). However, the clustering of biopsies into sites was not perfect. Hence we sought to quantify this trend by calculating the ratio of distances between pairs of biopsy samples from the same site and pairs of biopsy samples from different sites. This ratio was lower than expected for each of the subjects individually (*P* = 0.06 for *AA* and *P* = 0.11 for *NANA*, based on 50,000 permutations), and was significantly lower when considering both subjects together (*P* = 0.04 by Fisher's test). Thus, B cell clones display a tendency to expand locally within bronchial mucosa.

**Figure 8:**
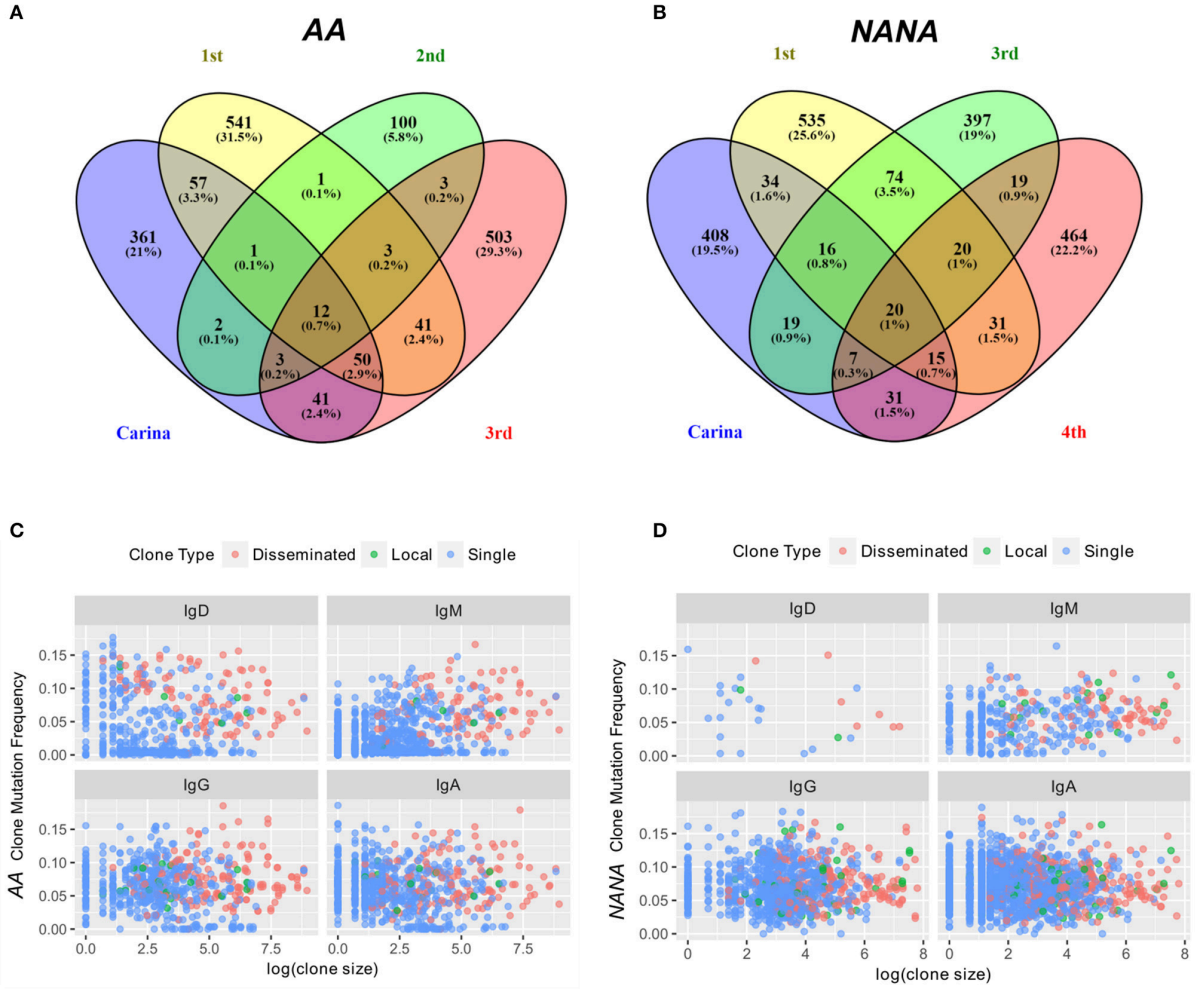
Some expanded clones have clonal members found in four sites within the bronchial mucosa. The numbers of clones overlapping between any combination of the four different bronchial mucosal sites sampled (carina and 1st to 3rd or carina and 1st, 3^rd^, and 4th generation lower lobe, respectively) were determined along with the number of clones contained in a single site for **(A)** the asthmatic subject *AA* and **(B)** the healthy subject *NANA*. The percentages refer to the percentages of the total repertoire from the bronchial mucosa of each individual. 0.7% (*AA*) and 1% (*NANA*) of all clones were found in all four sites, but the majority of the clones were found in only one site. Clones were divided into three groups (i) clones found in one biopsy only (single, blue dots), (ii) clones found in two to three biopsies from the same site (local, green dots) and (iii) clones found in at least two biopsies from at least two different sites (disseminated, red dots). For each of these categories and each of the four isotypes, the average clone mutation frequency was plotted against the log of the clone size for **(C)** the asthmatic subject *AA* and **(D)** the healthy subject *NANA*. In both individuals the disseminated clones appear, on average, to be larger and more mutated than the single clones with the less frequent local clones falling somewhere in the middle. No differences were found between the four isotypes analyzed.

To further investigate migration within the bronchial mucosa, we categorized clones into those found in only one biopsy (single), clones found in at least two biopsies from the same site in the bronchial mucosa (local) and clones found in at least two more distal sites (disseminated). Despite the bias toward local expansion, many clones were found at multiple sites in the bronchial mucosa (Figure 5) and we found that 20–30% of all clones were disseminated (data not shown). There was little variation in the degree of dissemination between the four sites and between the two individuals. Some clones spanned all possible combinations of sites, with ~1% of clones disseminated through all sites (Figures 8A,B). Sequences from the local clones were less mutated and of smaller clone size compared to sequences from disseminated clones, although it should be noted that wide ranges of mutation frequencies and clones were observed in both groups (Figures 8C,D). Local clones were intermediate in terms of size and mutation frequency. This was true for both *AA* and *NANA*, and suggests that B cell migration throughout the bronchial mucosa is associated with clonal expansion and SHM.

### Bidirectional travel between the bronchial mucosa and peripheral blood

**Figure 9:**
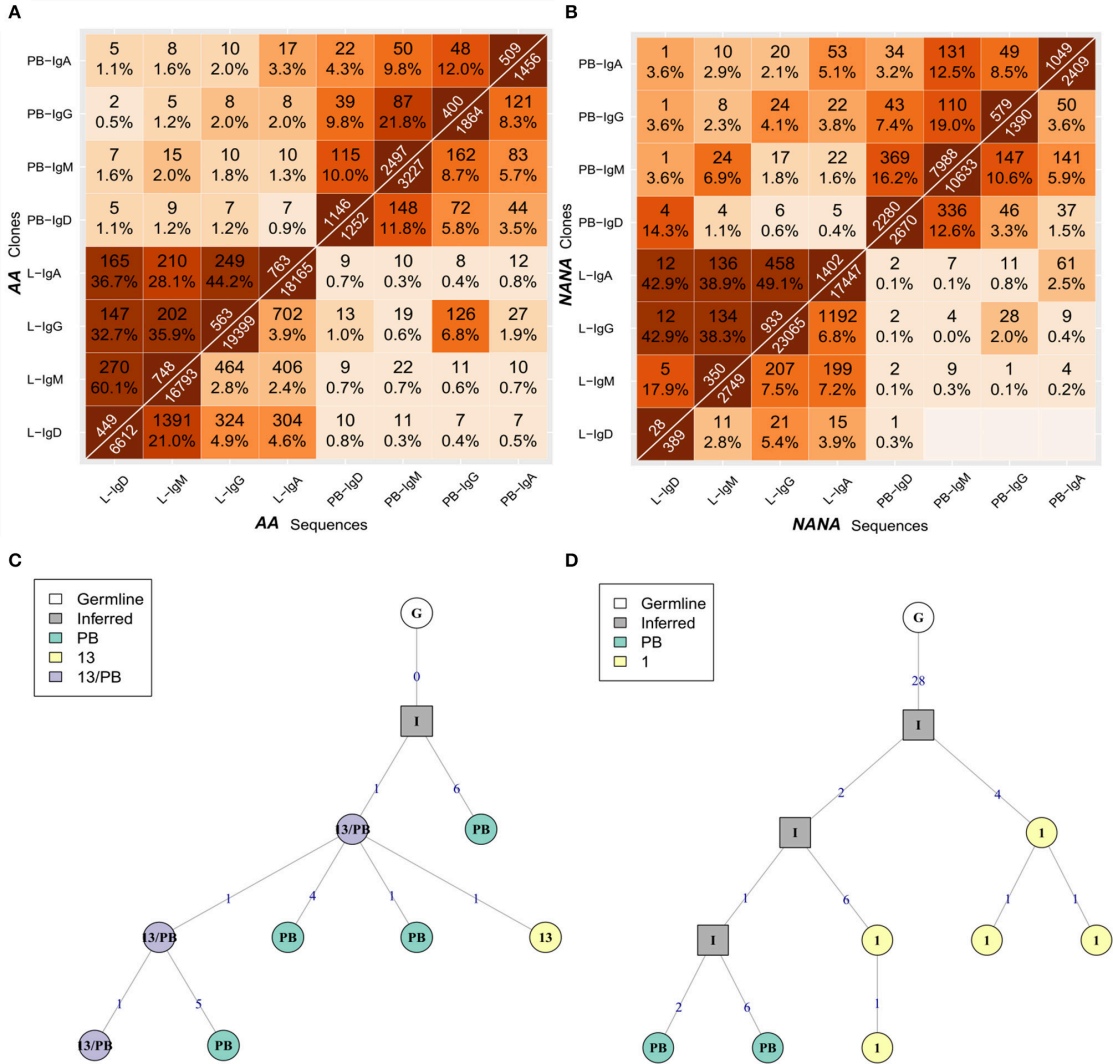
B cells of all isotypes can travel between the bronchial mucosa and peripheral blood. For each of the two subjects **(A)** the asthmatic patient *AA* and **(B)** the healthy subject *NANA*, the bronchial mucosal-derived clonal sequences were pooled and split into isotypes. For all pairs of isotype-specific bronchial mucosa and isotype-specific peripheral blood samples, the numbers and percentages of overlap between clones (top triangle) and unique sequences (bottom triangle) were computed as in Figure 3. The lower left hand quadrant shows the overlaps between all pairs of isotype-specific bronchial mucosal samples, the upper left hand quadrant shows the overlaps between all pairs of isotype-specific mucosal and peripheral blood samples at clonal level, the upper right hand quadrant shows the overlap between all pairs of isotype-specific peripheral blood samples and the lower right hand quadrant shows the overlaps between all pairs of isotype-specific mucosal and peripheral blood samples at the unique sequence level. **(C)** A representative clonal lineage tree from *AA* containing IgM sequences from peripheral blood (PB, green), biopsy 13 (13, yellow), and both (13/PB, purple). **(D)** A lineage tree for a heavily mutated IgA clone from *AA* with sequences from both peripheral blood (PB, green) and biopsy 1 (1, yellow). Square nodes with an “I” label indicate inferred sequences.

Having determined that many B-cell clones are disseminated throughout the bronchial mucosa, we next sought to determine the relationship between the bronchial mucosa and peripheral blood compartments. All samples showed good sequencing coverage (Figures S3A,B) and we found that all biopsy samples, with the single exception of AB12, shared at least one clone with the peripheral blood (Figures 5A,B). These shared clones were sometimes found only in one biopsy but more frequently in multiple sites within the bronchial mucosa (data not shown). This connectivity between the bronchial mucosa and blood involved all isotypes (Figures 9A,B, upper left and lower right quadrants), with the sharing greatest for clones and sequences of the same isotype. This was particularly apparent in *NANA*, with same-isotype overlaps ranging from 4.1% (24 of 579 clones) of PB (peripheral blood)-IgG related to L (bronchial mucosa)-IgG to 14.3% (4 of 28 clones) of L-IgD related to PB-IgD. In *AA* the highest degree of clonal overlap between peripheral blood and bronchial mucosa also involved the same isotype in both compartments, i.e., 3.3% (17 of 509) of PB-IgA clones related to L-IgA. Cross-compartmental overlap between different isotypes was lower in both individuals, ranging from 0.4% (5 of 1402 L-IgA clones related to PB-IgD) to 3.8% (22 of 579 PB-IgG clones related to L-IgA) in *NANA* and 0.5% (2 of 400 PB-IgG clones related to L-IgD) to 2.0% (10 PB-IgA clones related to L-IgG) in *AA*. Considering the entire repertoire, the fraction of clones shared was much higher between individual biopsy samples than between peripheral blood and biopsies (Figures 5A,B, 9A,B). In *AA* the highest degree of overlap was observed between IgM and IgD from the bronchial mucosa (60.1%: 270 of 449 of L-IgD related to L-IgM) and in *NANA* almost half of the bronchial mucosal IgG clones overlapped with IgA (49.1%: 458 of 933 L-IgG related to L-IgA). In general, there was a high degree of overlap between all isotypes within the bronchial mucosa (28-60% at the clonal level and 2.4–21% at the sequence level requiring total sequence homology) (Figures 9A,B, lower left quadrants). Overall, this analysis clearly demonstrated that B cells of all isotypes can travel between the bronchial mucosa and the peripheral blood in both asthmatics and healthy individuals.

To investigate the directionality of migration, we analyzed lineage trees for the 34 clones from *AA* and 55 from *NANA* with sequences in both the bronchial mucosa and the peripheral blood. We reasoned that if B cell migration between the two compartments is unidirectional, then the sequences from either the bronchial mucosa or the peripheral blood would fall consistently closer to the top node of the tree (i.e., would have undergone less mutation). Manual inspection of the lineage trees showed that in some clonal trees the least mutated sequence was from the peripheral blood and in others from the bronchial mucosa. Indeed, in some cases the least mutated node contained a sequence found in both peripheral blood and the mucosa (example in Figure 9C). This was true for both *AA* and *NANA*. We also established that mucosal and blood members in each of these shared clones had similar mean mutation frequencies (Figure 3C). Taken together these data suggest that B cells migrate between the bronchial mucosa and peripheral blood in a bidirectional manner.

### IgD-only B cells are prevalent in the asthmatic bronchial mucosa

**Figure 10:**
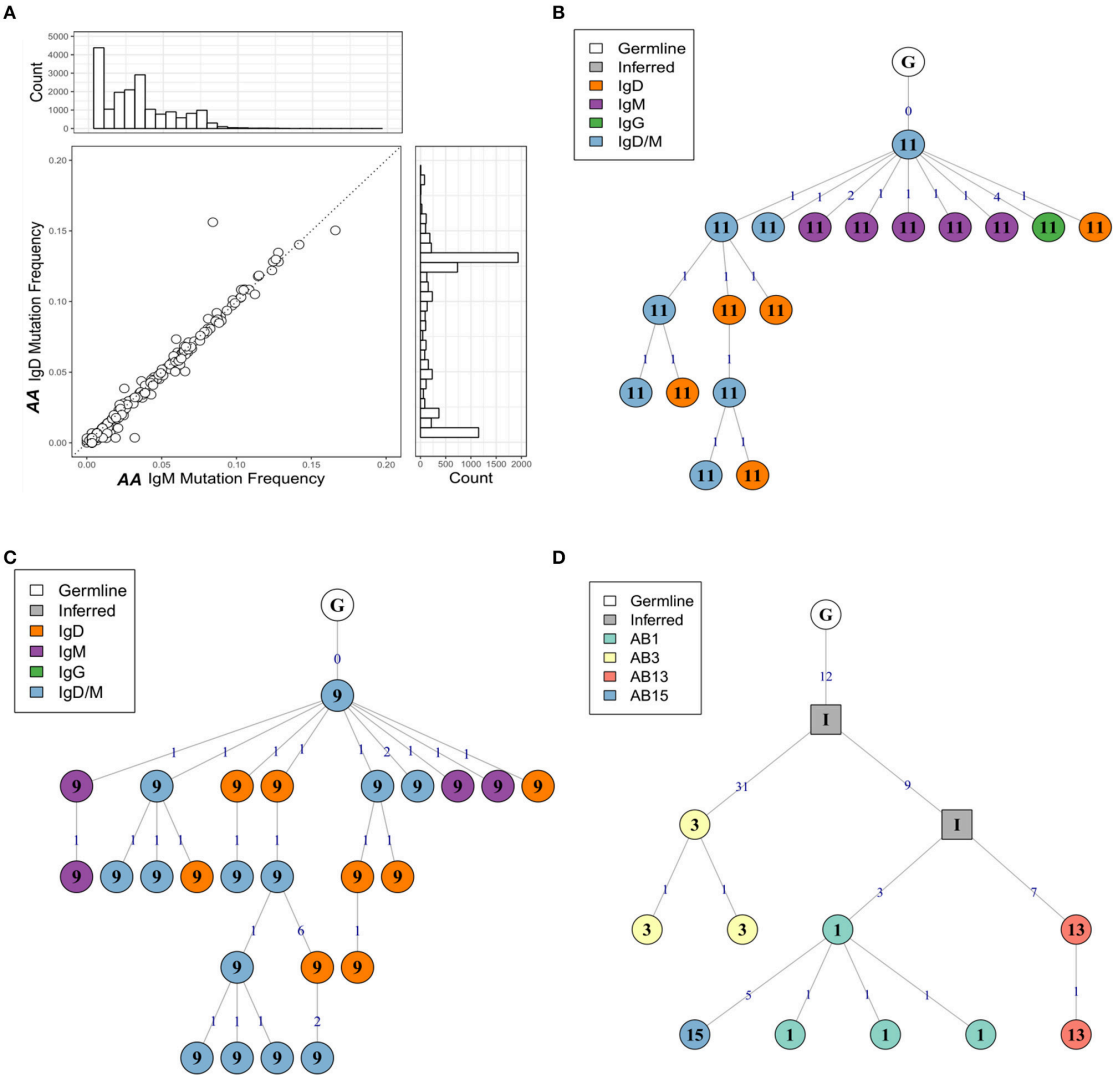
The mutation frequencies of IgD and IgM sequences from the same clone are highly correlated**. (A)** For each clone containing both IgM and IgD sequences from the atopic asthmatic *AA* bronchial biopsies, the median mutation frequency was calculated separately for IgM sequences and IgD sequences. Each circle represents one clone. The histogram on the right shows the mutation frequency for all IgD sequences from the bronchial biopsies and the histogram on top the mutation frequency for all IgM sequences for the bronchial biopsies. Lineage trees for two IgD/M clones from *AA* biopsies 11 **(B)** and 9 **(C)** showing dual IgD/M nodes (blue), along with separate IgD (orange) and IgM (purple) nodes. **(D)** A heavily mutated and expanded IgD-only clone with members from *AA* biopsies 1 (light green), 3 (yellow), 13 (red), and 15 (blue). Square nodes with an “I” label indicate inferred sequences.

As noted earlier, B cells in bronchial biopsies from *AA* contained a high proportion of IgD sequences, whereas IgD was rarely seen in the mucosa of *NANA* (Figures 1A,B). Furthermore, some of these tissue IgD sequences were highly mutated, with a mean mutation frequency (6.7%) similar to that of IgG (7.1%) and IgA (6.5%) (Figure 3A). The IgD sequences appeared, however, to segregate into two groups with low and high mutation frequencies (Figure 3A). A special subset of IgD-only cells has been described in the upper respiratory tract (25). These cells have a high mutation frequency (26, 27), and have lost the ability to co-express IgM and IgD through deletion of the IgM region in a class switch recombination event utilizing a cryptic switch region upstream of the IgD constant region (28). To investigate the possibility that some of the IgD sequences found in the bronchial mucosa of *AA* might originate from such IgD-only cells, we divided the IgD sequences into two groups—sequences from clones that contained both IgD and IgM sequences (IgD/M) and sequences from clones that contained IgD but no observed IgM sequences (IgD). In IgD/M clones, we found that the mutation frequency of IgD and IgM sequences from the same clone was highly correlated (Figure 10A), suggesting that these clones represent cells expressing both IgD and IgM, even though we did not observe both isotypes for every single sequence, presumably as a result of incomplete sampling and sequencing depth. The clones spanned a range of mutation frequencies but only a few fell in the highly mutated range that was prevalent for the IgD sequences (Figure 10A—histogram on the right).

**Figure 11:**
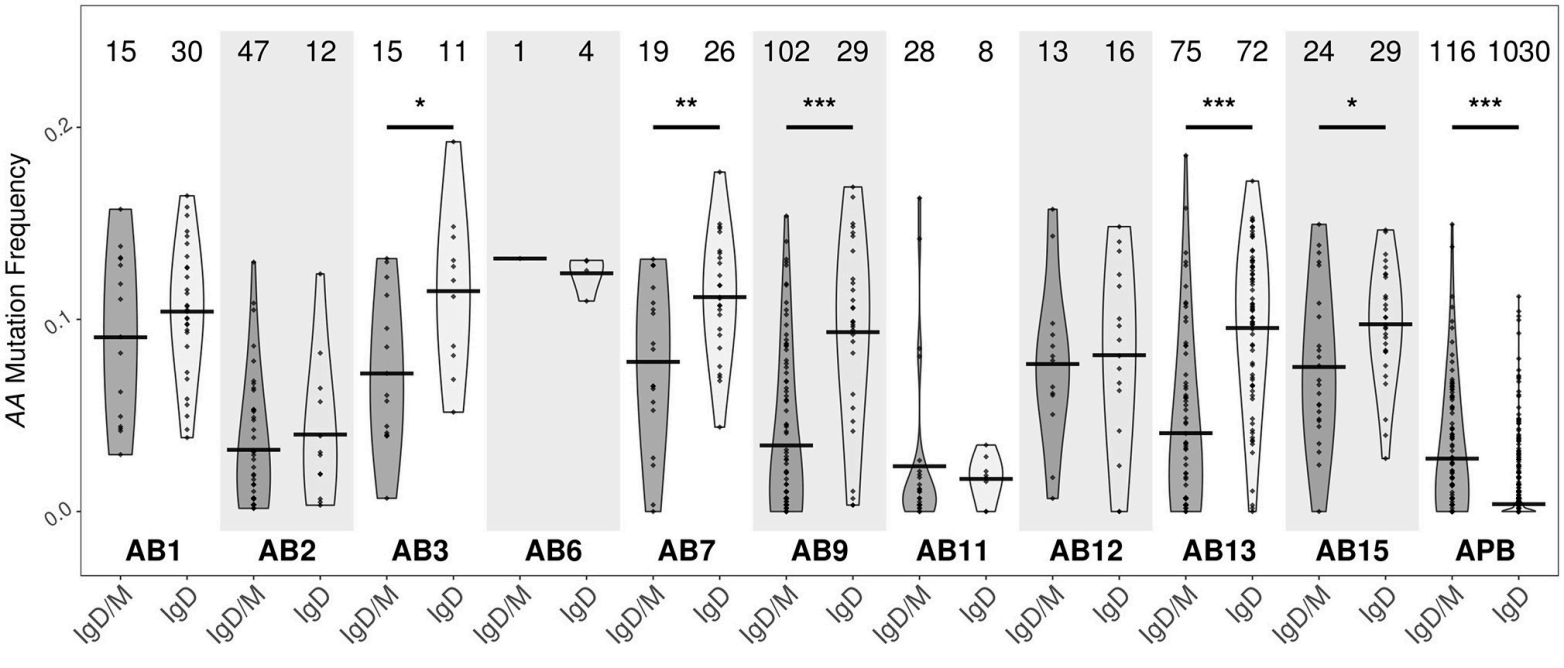
IgD-only clones from the bronchial biopsies are highly mutated. Clones containing IgD sequences from each of the 10 bronchial mucosal biopsies and peripheral blood from the asthmatic patient *AA* were divided into two groups according to whether the clone also contained IgM sequences (IgD/M) or not (IgD). Within each group, the mutation frequency of a clone (points) was summarized as the median mutation frequency of IgD sequences. Horizontal lines indicate the mean mutation frequencies for the clones in the given sample category. The numbers of clones in each sample are shown above each violin. *P < 0.05, **P < 0.01 and ***P < 0.001 indicate a significant difference between the median mutation frequencies for IgD in IgD/M and IgD in IgD-only clones from the given sample.

With few exceptions, the mutation frequencies of the IgD-only bronchial mucosal clones were significantly higher than those of the IgD/M clones (*P* < 0.05) (Figure 11). Analysis of the mutation patterns in these sequences revealed that they corresponded to hot- and cold-spot preferences typical of SHM (Figure S2B) suggesting that the high mutation load had been generated by normal SHM. The selection strength also appeared to be similar in the two cell subsets (Figure 4A). Figures 10B,C show the clonal trees from two IgD/M clones, both found in one biopsy only (AB11 and AB9, respectively) and both containing three major types of nodes: (1) IgD only, (2) IgM only and (3) both IgD and IgM. In both trees, the least mutated node contained a germline sequence found as both IgM and IgD. All sequences within the trees had very few mutations. In contrast, the tree in Figure 10D is from an IgD-only clone. This clone contained heavily mutated sequences found in four different biopsies (AB1, AB3, AB13, and AB15). These analyses further strengthen the conclusion that the bronchial mucosa from the asthmatic subject contains highly mutated, IgD-only clones.

**Figure 12:**
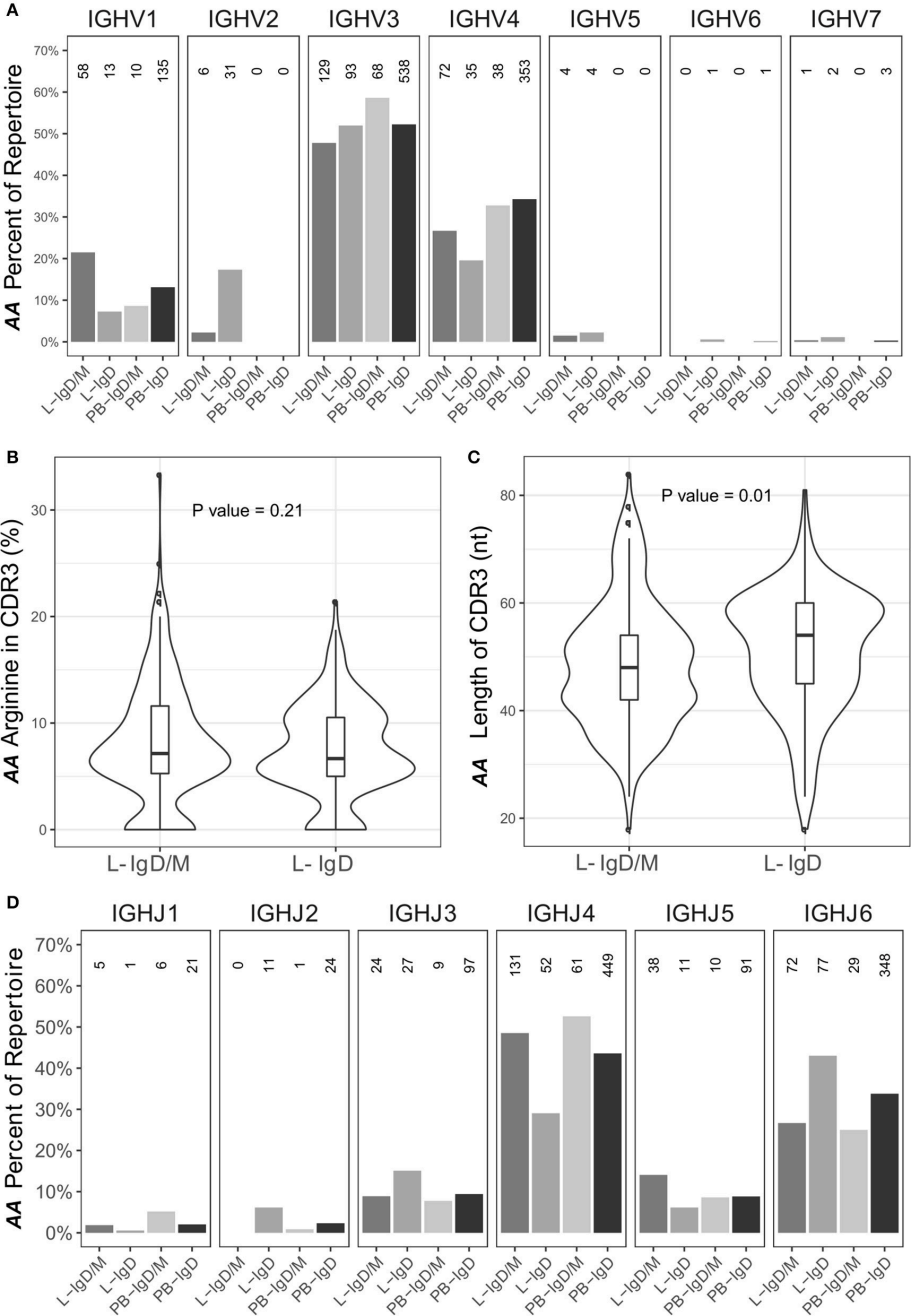
The IgD-only repertoire is distinct from the IgD/M repertoire. Clones containing IgD sequences from the asthmatic patient *AA* were divided into two groups according to whether the clone also contained IgM sequences (IgD/M) or not (IgD). **(A)** The VH gene usages for IgD clones from the two groups from the bronchial mucosa (L) and peripheral blood (PB) were calculated and plotted as described for Figure 1E with the numbers of clones in each category shown above each bar. For each of the two groups, the **(B)** arginine content and **(C)** length of the CDR3 region, as defined by IMGT, was calculated. Horizontal lines indicate the median and the box the 25–75th percentiles. **(D)** The JH gene usage for IgD clones from IgD/M and IgD-only clones was calculated and plotted as described for Figure 1E.

Certain IgD-only cells have been reported to be auto-reactive (29), to have specificity against respiratory pathogens (30) or to be driven by superantigen (31). In view of this, to further characterize the sequences from the IgD-only clones, we analyzed their VH gene usage as described in Figure 1 (Figure 12A). In peripheral blood we observed no significant difference between the IgD-only and IgD/M groups, but in bronchial mucosa VH1 was statistically significantly under-represented in the IgD-only clones compared with the IgD/M clones (*P* = 2.2e^−16^ by Fisher's test), whereas VH2 was over-expressed (*P* = 2.2e^−16^ by Fisher's test). VH3 and VH4 were the most frequently used VH genes as seen for the other immunoglobulin isotypes (Figure 1E) and with these gene families we observed no significant disparity of expression in the IgD/M and the IgD-only clones. However, when we restricted the analysis to the VH3-30 gene, which has previously been reported to be over-expressed by IgD-only cells (31), we observed that VH3-30 was indeed significantly over-expressed in the bronchial mucosal IgD-only B cell clones compared with both the mucosal IgD/M clones (*P* < 0.001 by Fisher's test) and the peripheral blood IgD-only clones (*P* < 0.001 by Fisher's test) (data not shown). Genes from the VH5, VH6, and VH7 families were expressed in only a tiny minority of the clones, with VH5 detected only in clones from the bronchial mucosa in agreement with our finding of elevated VH5 usage in the mucosa compared with the peripheral blood. Thus, IgD-only cells in the bronchial mucosa showed a distinct pattern of VH gene usage.

Sequences from IgD-only cells have also been reported to have longer CDR3 regions with more arginine compared to other isotypes as well as to over-express JH6 (29). We did not find a higher percentage of arginine in the CDR3 region of the IgD-only clones compared to IgD/M clones (*P* = 0.21). We did however find that the IgD-only clones had statistically significantly longer CDR3 regions (*P* = 0.01) and statistically significant over-expression of JH6 (*P* = 2.5 x 10^−4^) (Figures 12B–D, respectively). As JH6 is the longest of the JH gene segments, we were interested to see whether these long CDR3 regions reflected the over-usage of JH6. This was not the case, since we found that for clones using JH1-5, IgD-only cells also had significantly longer CDR3 regions than IgD/M clones (*P* = 0.001) (data not shown). Interestingly, IgD-only clones using JH6 did not have significantly longer CDR3 regions compared to IgD/M clones (data not shown), suggesting that IgD-only clones are selected for long CDR3s *per se*. Thus, the IgD-only clones identified in our dataset appeared to be from a special subset of B cells displaying a high degree of SHM, long CDR3 regions and increased usage of JH6 and the VH3-30 gene, in line with previous reports on IgD-only cells. In terms of dissemination, the IgD clones conformed with the patterns seen with the other isotypes in that most clones were found in one biopsy only (76%), a few were found to be locally expanded (2%) and some were disseminated between distant sites (22%). None of the IgD-only clones had clonal members found in the peripheral blood.

## Discussion

B cells and the antibodies they produce constitute a significant plank of the mucosal host defense barrier against the panoply of environmental assaults to which they are exposed. Study of the construction of this barrier is a considerable challenge: our knowledge of the extent and genesis of the airways mucosal B-cell antibody repertoire is almost non-existent. This is partly because the total surface area of the mucosa is very large, while ethical and practical constraints dictate that only small areas can be sampled at any one time. The new and rapidly evolving technique of Adaptive Immune Receptor Repertoire Sequencing (AIRR-Seq) affords new opportunities to characterize the extent and evolution of the local immunoglobulin repertoire from small samples, and this study represents the first application of AIRR-Seq to study B cells in relatively small individual biopsies from the human bronchial mucosa.

We have here characterized the immunoglobulin repertoire in B cells from contemporaneous samples of the bronchial mucosa and the peripheral circulation in two subjects, one asthmatic, *AA*, as a prototypical airways mucosal inflammatory disease, and one healthy subject, *NANA*. Previous studies pointed to geographical variability within the bronchial mucosa in the numbers of B cells and antibody specificities (32) and appearance of B cell clusters in some of the biopsies (33). We therefore decided to examine multiple regions of the mucosa between the carina and the 4th generation bronchi of the lower lobe of the right lung (Figure 7), with several samples from each of those sites. In total we analyzed 10 biopsies from four distinct sites from each individual. For all biopsy samples the sequence coverage was very high (Figure S3) suggesting that we could identify the antibody sequences from most of the B cells from each biopsy. AIRR-Seq showed that the biopsies contained between 50 and 500 B cell clones (Figure 1), in line with the B cell numbers estimated in our immunohistochemical and flow cytometric analyses of dissociated bronchial biopsies [(33) and unpublished results].

The repertoires from both the bronchial biopsies and the peripheral blood exhibited diversity in the expression of different isotypes, VH gene usage and SHM (Figure 1). While the VH gene usage was broadly similar in the bronchial mucosa and peripheral blood of both subjects, resembling that previously reported from blood in the normal population (18), we did observe over-expression of the VH5 family genes in the mucosa compared to peripheral blood in both subjects, confirming previous reports in asthma (17, 34) and in other atopic diseases: rhinitis (5, 35) and atopic dermatitis (36).

It has been suggested that the over-expression of VH5 by the B cells of the bronchial mucosa could reflect the activity of a local B cell superantigen, possibly a common virus or commensal bacterium (17). There is some precedent for this hypothesis as Domiati-Saad et al. have shown that *Staphylococcus aureus* enterotoxin D (SED) and SEA can act as B cell superantigens by rescuing B cells utilizing VH4 and VH3 genes, respectively, from apoptosis *in vitro* (37, 38). We have also previously identified one such superantigen, SEE, in nasal polyps from patients with chronic rhinosinusitis (39). The blood IgE compartment in atopic asthmatic children has previously been found to exhibit a classical, antigen-driven response inconsistent with a superantigen driven response (40), whereas the IgE compartment in blood of young children with atopic dermatitis, does indeed display skewed VH gene usage and low levels of somatic hypermutation, which may be interpreted as signs of activation by a superantigen (41). The superantigen hypothesis has also been invoked to explain a skewed VH gene usage in IgD-only cells. Seifert *et al*. observed that over 30% of peripheral blood IgD-only cells and 50% of tonsillar IgD-only cells utilized VH3-30 (31). We also observed a strong bias toward usage of the VH3-30 gene (46% of bronchial mucosa IgD-only sequences), along with the statistically significant over-expression of VH2 and under-expression of VH1 family genes in IgD-only compared to IgD/M sequences in *AA* (Figure 12), supporting the hypothesis that superantigens or antigens expressing a repeating (e.g. carbohydrate) epitope may be involved in the activation of IgD-only cells.

After the various quality control steps we could not detect IgE transcripts in any of the bronchial mucosal samples from the asthmatic patient *AA* and only one from the healthy subject *NANA*, although they were found in blood. This may reflect our earlier experience with irreproducibility in analyzing mRNA from small bronchial biopsies, which we attributed to the relatively low copy number of expressed epsilon mRNA in the biopsies (unpublished results). We found that the IgE primers used for amplification had very high homology with IgG. This increases the risk of mispriming and subsequent quality control failure, especially with low mRNA copy numbers, whereas amplification of the higher numbers of epsilon transcripts in the peripheral blood samples would be possible. The number of detectable IgE sequences and clones in the peripheral blood was higher in the healthy subject than the asthmatic patient, possibly reflecting the higher total serum IgE concentration in the former (see Supplementary Materials). In both subjects the peripheral blood IgE repertoire displayed a broad VH-gene usage in line with previous reports of IgE repertoires in both atopic and non-atopic individuals (42–44) and in allergen-specific IgEs (45).

We noted a significantly wider geographical variability of the total immunoglobulin repertoire in the bronchial mucosa of the asthmatic patient compared with that of the healthy subject. This was both in terms of isotype distribution, mean mutation frequency, and measures of diversity (Shannon and Simpson indexes) (Figures 1, 2). In contrast, the overall size of the repertoire was significantly less diverse (Figure 2), which we interpret as reflecting ongoing B cell clonal amplification within the inflamed mucosa of the asthmatic patient. This is supported by our finding of higher numbers of unmutated and presumably naïve IgM/IgD cells in the mucosa of the asthmatic compared with the healthy subject (Table 1 and Figure 1A,B), which also points to a more focused subset of B cells in the bronchial mucosa of the asthmatic that are continuously activated, resulting in larger clones and a oligoclonal response. The drivers for this oligoclonal response remain to be clarified. Intuitively, a host of environmental factors may influence the shaping of the immunoglobulin repertoire of B cells at a mucosal surface exposed to the external environment in ways that are not yet well defined. In the case of asthma these might include, amongst others, the effects of topical medications, exposure to environmental proteins including aeroallergens, intercurrent respiratory tract infections, severity and longevity of disease and exposure to environmental pollutants, including cigarette smoke. Further dissection of the influences of these various factors will require further studies with carefully chosen patient and control subject cohorts.

Remarkably, we uncovered clear evidence that B cell clones from any one site in the bronchial mucosa can be widely disseminated within the mucosa and further that the more a given clone has expanded and mutated, the more likely it is to have migrated to a remote site within the bronchial mucosa (Figure 8). In both subjects we found that the B cells from within each of the four distinct sites analyzed tended to have a higher degree of clonal overlap compared with those in biopsies from more distal sites (Figure 7). This suggests that there is local proliferation, SHM and migration of B cells *in situ* as well as widespread trafficking of B cells between different sites in the mucosa, a potentially vital protective defense mechanism that may have been established in evolution. The potential significance of this trafficking is that a selected B cell clone directed against an antigen originating at one site in the bronchial mucosa may sensitize the entire bronchial mucosa.

We have previously shown that expression of the components required for B cell SHM and class switch recombination, such as AID, can be detected in the bronchial mucosa itself (2). It is also possible that these events might occur, at least partly, at discrete sites such as inducible bronchial associated lymphoid tissue, iBALT, identifiable in the bronchial mucosa of at least some asthmatics, but more difficult to find in healthy controls (46–48). This may in turn reflect the observation that the airways of asthmatics are more susceptible to a range of environmental assaults, most notably bacterial and viral infections, which may promote the formation of the germinal center-like structures in iBALT that may persist for months after the initial assault, causing chronic inflammation (46, 48). If B cell maturation takes place in iBALT, one can envisage that the B cells may migrate directly both to local and more distal sites within the lung through the local blood or lymphatic networks. Lymphatic vessels are known to surround iBALT, although their exact function is unknown (48). In future studies it would be interesting to compare the B cell antibody repertoire in the bronchial mucosa of both lungs with that of the peripheral blood, as this would likely throw more light on the pathways of B cell migration in the bronchial mucosa, and particularly whether it occurs predominantly locally or via the lymphatics/systemic circulation. Nevertheless, the data presented here are similar to those obtained in the gastrointestinal tract by Holtmeier et al. who observed expanded IgA clones from multiple and distant sites across the colonic mucosa in four healthy individuals. As in the present study in the bronchial mucosa, a minority of these clones was also found in blood (8).

It has been demonstrated that the B cell repertoires in the gut and airways mucosa are distinct (9), and that the repertoires from different sites in each organ appear to show greater overlap than those between the organs and the peripheral blood. This could reflect the possibility that local migration of B cells within an organ or mucosal surface does not involve the peripheral blood. Alternatively, it may reflect the fact that the blood contains a mixture of B cell clones activated in various organs and exposed to a distinct range of environmental assaults, as well as others that may originate in the germinal centers of systemic lymph nodes. It would follow that the B cell repertoire in a sample of peripheral blood is unlikely to represent the total repertoire in blood, so that even some of the largest clones in the mucosal surfaces of organs may be too diluted to appear in a small sample of blood. Keeping these points in mind, our observed degree of overlap of the B cell clones in different regions of the bronchial mucosa (Figures 5, 8) may result from the trafficking of clones through the blood and homing back to various sites in the tissue, and/or emigration from, then re-entry into the mucosa as they pass by distal branches of the draining lymphatics before exiting the lung. The latter scenario would support our finding that trafficking between the bronchial mucosa and peripheral blood is bi-directional (Figure 9) and without a clear indication of the initiation site for B cell expansion and maturation. In contrast to our findings, a study in multiple sclerosis using AIRR-Seq on paired tissue samples suggested that the progenitors of the expanded, antigen-experienced B cell clones sequenced from the brain originated in the draining cervical lymph nodes. The clones subsequently migrated to, and potentially further diversified in more distant regions of the nervous system (6). The mechanism of B cell dissemination in the bronchial mucosa may differ from that in the brain given that the B cells are continuously exposed to environmental or endogenous antigens and that the mucosa is supplied with a rich network of blood vessels and populated with its own lymphoid tissue. The Stern study (6) does, however, demonstrate that B cells traffic between tissue and periphery, which is in line with the results of the present study.

The precise role of IgD antibody remains elusive (30). IgD is expressed both by IgM^+^IgD^+^ cells by alternative splicing of the pre-mRNA and also by isotype switched, IgD^+^ only cells (26, 30). The latter are sparse in the circulation and the bone marrow, but more frequent in germinal centers and the extra-follicular areas of, for example, the nasal mucosa and tonsils (27, 30). Here we provide the first concrete evidence for the presence of IgD-only B cells within the *bronchial* mucosa. We identified the IgD-only sequences based on a greater degree of SHM than those from clonal families that included IgM sequences (Figure 11). A high propensity for SHM in IgD-only cells has previously been described as an important feature of this cell subset (26, 27, 31).

We also found that the sequences from IgD-only cells had significantly longer CDR3 regions and over-expressed VH3-30 and JH6 (Figure 12), in line with previous reports (29, 31). Long CDR3 regions are often associated with auto-reactivity, and Koelch *et al*. found that some IgD-only cells produced antibodies against nuclear antigens, single- and double-stranded DNA and/or human epithelial type 2 cells (29). It has also been shown that some IgDs have specificity for respiratory pathogens such as *Haemophilus influenzae* types a and b and *Moraxella catarrhalis* (30), and that the serum concentrations of allergen-specific IgD correlate with those of allergen-specific IgE in atopic subjects (49). Circulating, secreted IgD can bind to basophils and mast cells through an uncharacterized receptor, and cross-linking of this IgD on the basophils results in the release of pro-inflammatory mediators including TNF-α and IL-1β, and B cell activating factors including IL-4, IL-3, BAFF, and APRIL (28, 30).

It is reported that IgD-only cells cannot undergo class switching to downstream isotypes in the immunoglobulin heavy chain locus (26). This is supported by our data where fewer than 10% of IgD-only clones contained sequences of the IgA or IgG isotype, and furthermore analysis of the clonal trees suggested that class switch to the downstream isotypes might have occurred before the loss of IgM expression. Nevertheless, we hypothesize that highly mutated bronchial mucosal IgD from IgD-only cells may be involved in asthma pathogenesis by increasing the diversity of the total immunoglobulin repertoire that, for example, could play a role in augmenting local mucosal inflammation. This could be through cross-linking of specific IgD on the surface of mast cells and/or basophils, resulting in the release of pro-inflammatory mediators as described above. With this in mind, it is interesting that we found high numbers of IgD-only B cells only in the bronchial mucosa of the asthmatic subject. Very few IgD sequences were found in the B cells in the bronchial mucosal biopsies of the healthy subject, but those that were found were typically highly mutated and from IgD-only clones (80% bronchial mucosa IgD), which reveals that IgD-only B cells can also be found in the non-diseased state. We suggest that either the numbers may be too small to cause damage or, in line with the hypothesis from Chen et al. (28, 30), that their roles may lie more in enhancing mucosal immunity than in disease pathogenesis. Our results echo a recent study in which elevated numbers of IgD^+^ plasmablasts and increased soluble IgD was found in nasal tissue in patients with chronic rhinosinusitis compared with controls (50).

In summary, in this manuscript we show that AIRR-Seq is a powerful tool for analyzing the B cell repertoire from individual biopsies from multiple sites of the bronchial mucosa. Conventional methods such as immunohistochemistry can be used to enumerate the various cell types in the tissue, but only AIRR-Seq can provide information about clonal relationships, SHM and VH gene usage, relevant for understanding the diversity and focus of the local antibodies. Our analyses have produced important and novel information concerning a high degree of connectivity of clonal B cell development in widely spaced regions of the bronchial mucosa and bi-directional travel of cells between mucosa and the peripheral blood. Thus we here provide proof of principle that AIRR-Seq can unlock data which will enable understanding the initiation and propagation of antibody responses at environmental interfaces such as the respiratory mucosa, and an opportunity to investigate variability in this response which may be linked with phenotypes/genotypes of disease severity and longevity, modification by therapy and environmental exposure. Our analyses also reveal that the B cell repertoire in the bronchial mucosa of the asthma patient is less diverse and hence more focused, and contains more naïve cells than that of a healthy subject. To discover whether these characteristics are truly related to disease status, we are currently engaged in a study using AIRR-Seq as a highly sensitive method to characterize the B cell repertoire in mucosal tissue in a larger group of individuals. Finally, for the first time, we have identified sequences from IgD-only B cells in the lower respiratory tract mucosa and shown that AIRR-Seq can be used to characterize the repertoire properties of these elusive cells, affording new ways in which to discover the triggers and effector functions of IgD antibodies in the pathophysiology of asthma and potentially other airways mucosal diseases.

## Author contributions

LO-L and HJG designed the research; CJC and LO-L collected and processed the samples, respectively; HM, JC, JQZ, and SHK analyzed the data and produced the figures in discussion with LO-L and HJG; LO-L, HJG, SHK, and CJC wrote the paper.

### Conflict of interest statement

CJC has received fees for attending advisory boards from Chiesi and Mundipharma and lecture fees from GlaxoSmithKline. He has attended international conferences with Novartis and Boehringer Ingelheim. The remaining authors declare that the research was conducted in the absence of any commercial or financial relationships that could be construed as a potential conflict of interest.
