# Supplementary material for: Local Clonal Diversification and Dissemination of B Lymphocytes in the Human Bronchial Mucosa
Source: Front Immunol. 2018 Sep 7;9:1976. doi: 10.3389/fimmu.2018.01976 (PMC6137163; doi:10.3389/fimmu.2018.01976)
Supplement: Supplementary file 1 [file Data_Sheet_1.pdf]

Supplementary Material

Local clonal diversification and dissemination of B lymphocytes in the human bronchial mucosa

Line Ohm-Laursen*, §, Hailong Meng§, Jessica Chen, Julian Q Zhou, Chris J Corrigan, Hannah J Gould§§, Steven H Kleinstein§§

§ and §§ these authors contributed equally to the work

*** Correspondence:**
Line Ohm-Laursen
line.ohm-laursen@kcl.ac.uk

Supplementary methods

Participant characteristics

AA was a 65-year-old Caucasian man with a positive skin prick tests to mixed grass, house dust mite, *Alternaria* and *Aspergillus* and a confirmed diagnosis of asthma. His lung function was poor with a pre-bronchodilator FEV1 of 52% of the predicted value and 22% reversibility in response to bronchodilator. The patient was taking Seretide Evohaler® (fluticasone propionate 50 mg/puff, salmeterol xinafoate 25 mg/puff) one puff twice daily. *NANA* was a 42-year-old Caucasian female who was ostensibly healthy and had never suffered from asthma or symptoms of any other atopic disease. She had a negative skin prick tests to a panel of nine common aeroallergens. Her lung function was normal with a pre-bronchodilator FEV1 of 109% of the predicted value. Serum total IgE concentrations as measured by the ImmunoCAP® platform were 100 kU/l for *AA* and 181 kU/l for *NANA*. The latter is about 2 SD above the mean values in healthy, non-atopics (32), illustrating that non-atopic subjects may have elevated total circulating IgE in the absence of allergen-specific IgE. Both individuals were non-smokers.

Sequencing data processing

With data received from iRepertoire, the CSV output files were converted into FASTA and processed with the ImMunoGeneTics (IMGT)/High V-QUEST database for V(D)J assignments using the December 14, 2015 version of the IMGT gene database (12). The data were then processed with the Change-O v0.3.2 (13), Alakazam v0.2.5 (13), SHazaM v0.1.4(13), and other custom scripts through the R environment (14). Nonfunctional sequences were also removed from the data, as were potential chimeras identified as sequences with more than six mismatches in a 10-nucleotide stretch. Sequences were partitioned into clones with the "DefineClones" command in the Change-O package (13). Specifically, data were first separated into groups with the same VH gene, JH gene and junction length. The junction region is defined from the codon 104 encoding the conserved cysteine to codon 118 encoding phenylalanine or tryptophan. The distance between each sequence to its nearest sequence was then determined. A histogram of these distances was manually inspected to determine a Hamming distance (normalized by junction length) of 0.1 as a threshold for assigning clonal groups following single-linkage hierarchical clustering. After manual inspection of the number of duplicate identical sequences (copies) in the data, a threshold of 5 copies or greater was determined. All sequences that had fewer than 5 copies, as determined by iRepertoire, were filtered out of the data. Due to the low quantity of IgE sequences, all copies of IgE were retained in the dataset.

Isotype assignment

Based on the set of isotypes, sub-types and known alleles as listed in the IMGT database, we derived a list of isotype signature sequences from the IMGT CH region and JH segment consensus reference sequences (12) to accurately assign isotypes to each sequence (Supplementary Table 2). These signature sequences were found at the 5' end of constant region upstream of the constant region primers used by iRepertoire. The signature sequences were used to align against the tail sequences (JH segment end) using "MaskPrimers" in the pRESTO package (SR1) to make accurate isotype calls. Sequences with any mismatch between isotype signature sequences and the aligned sequence were discarded.

Calculation of mutation frequency

For each sequence, the mutation frequency was calculated as the number of mutations over the total number of positions that were classified by IMGT High V-Quest as VH and JH. The median value for all sequences belonging to a given clone was used to calculate the overall mutation frequency for that clone. We have adopted this approach to avoid skewing of the result by larger clones.

Generation of clonal trees

Sequences with the same isotype, junction length and VH and JH sequences from within the same clone of the same sample were treated as duplicated sequences. Duplicated sequences were combined and annotated by the function makeChangeoClone of Alakazam v0.2.5 (13). Lineage trees were inferred via maximum parsimony with PHYLIP version 3.69 (SR2). The analysis of lineage tree topologies was performed using standard graph traversal algorithm provided by the igraph R package version 0.7.1 (SR3) .

Selection analysis

The selection strength on clonal lineages was quantified with BASELINe (23) implemented in SHazaM (13), using the "HH_S5F" model (21) as the underlying somatic hypermutation targeting model from which expected mutation rates are derived and the local test statistic (24). Each isotype from a clonal lineage was represented by the most highly mutated sequence of that isotype present in the clone. The probability density functions (PDF) for the selection strength on the complementarity-determining region (CDR) and the framework region (FWR) were computed by unweighted convolution of the PDFs for individual clones corresponding to the respective regions.

Connectivity analysis

A clone count matrix was generated with each column associated with a biopsy and each row a clone. Entries in the matrix were given by the number of sequences in each clone normalized by the total number of sequences in the biopsy (*i.e.*, column normalized). Manhattan distances between every pair of biopsies were then calculated based on the normalized clone counts. Multidimensional scaling (MDS) was applied to these distances to visualize the relatedness of samples using R function "cmdscale" from package stats. To test whether the distances of biopsies from within the same lobe were significantly closer compared with the distances of biopsies from different lobes, we calculated the ratio of the average within-lobe distances and average between-lobe distances. A background distribution was generated by permuting the labels of the biopsy samples 50,000 times and recalculating this ratio. The P value was then calculated at the fraction of ratios from the background distribution that were less than or equal to the observed ratio.

Supplementary tables and figures

Supplementary Table S1 Numbers of sequences retained after each filtering and quality control step

	Sequences from iRepertoire	Match from IMGT	Functional sequences	Copy number >4 (>=1 for IgE)	After all filtering and QC steps
AA **Bronchial mucosa**[a]	2,539,379	2,539,054	2,424,747	115,531	109,763
AA **Peripheral Blood**	590,581	590,464	571,584	11,894	11,744
NANA **Bronchial mucosa**[a]	2,793,835	2,793,691	2,668,191	98,837	94,667
NANA ***Peripheral Blood***	858,616	858,393	829,683	23,161	22,754

AA: atopic asthmatic subject, *NANA*: non-atopic, non-asthmatic subject

a: All ten biopsies from the individual combined

Supplementary Table S2 Signature sequences for isotype identification

Isotype	Sequence
**IgD**	5’-GCACCCACCAAGGCTCC-3’
**IgM**	5’-GGGAGTGCATCCGCCCC-3’
**IgG1/2**	5’-GCCTCCACCAAGGGCCC-3’
**IgG3/4**	5’-GCTTCCACCAAGGGCCC-3’
**IgA**	5’-GCATCCCCGACCAGCCC-3’
**IgE**	5’-GCCTCCACACAGAGCCC-3’

Supplementary Figure S1

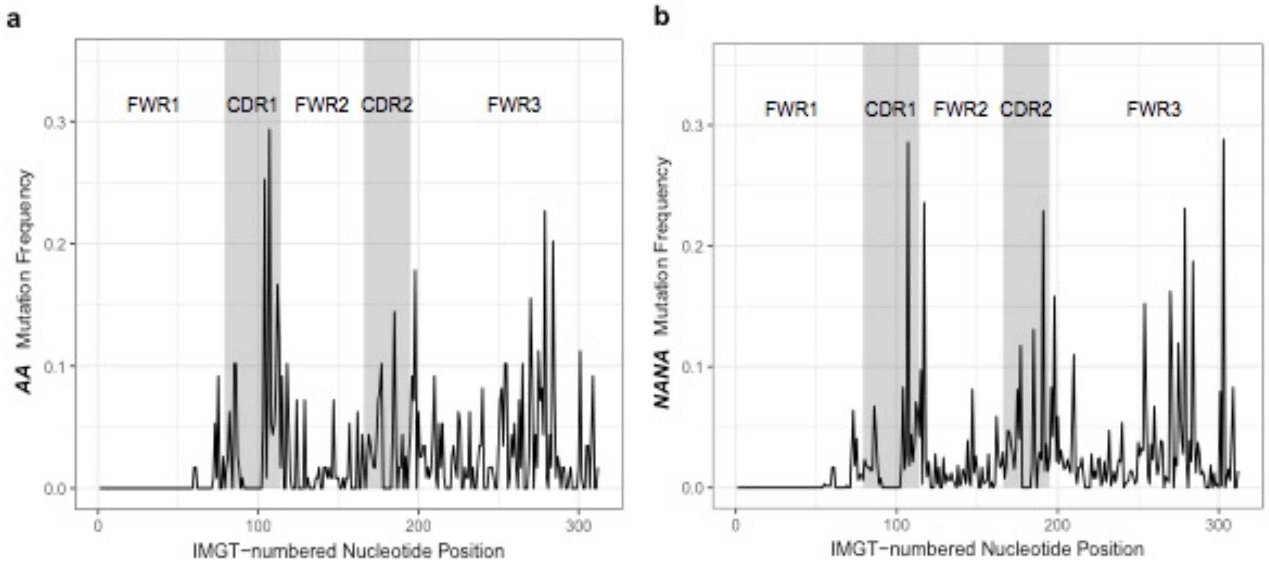

Supplementary Figure S1
The observed pattern of somatic hypermutation across the VH gene. The mutation frequencies per nucleotide across the length of the VH-region (IMGT numbering (SR4)) were calculated and displayed for (A) the asthmatic subject *AA* and (B) the healthy subject *NANA*. The grey shaded areas indicate the complementarity regions (CDR) and the white areas the framework regions (FWR). The signal starts in nucleotide position 50-55 depending on VH gene family since the PCR primers used for generating the sequencing products bound just upstream of this position. Mutation frequencies show the expected pattern with high frequencies in the CDRs and towards the end of FWR3, reaching almost 30% in some positions, and lower frequencies in the rest of the FWRs.

Supplementary Figure S2

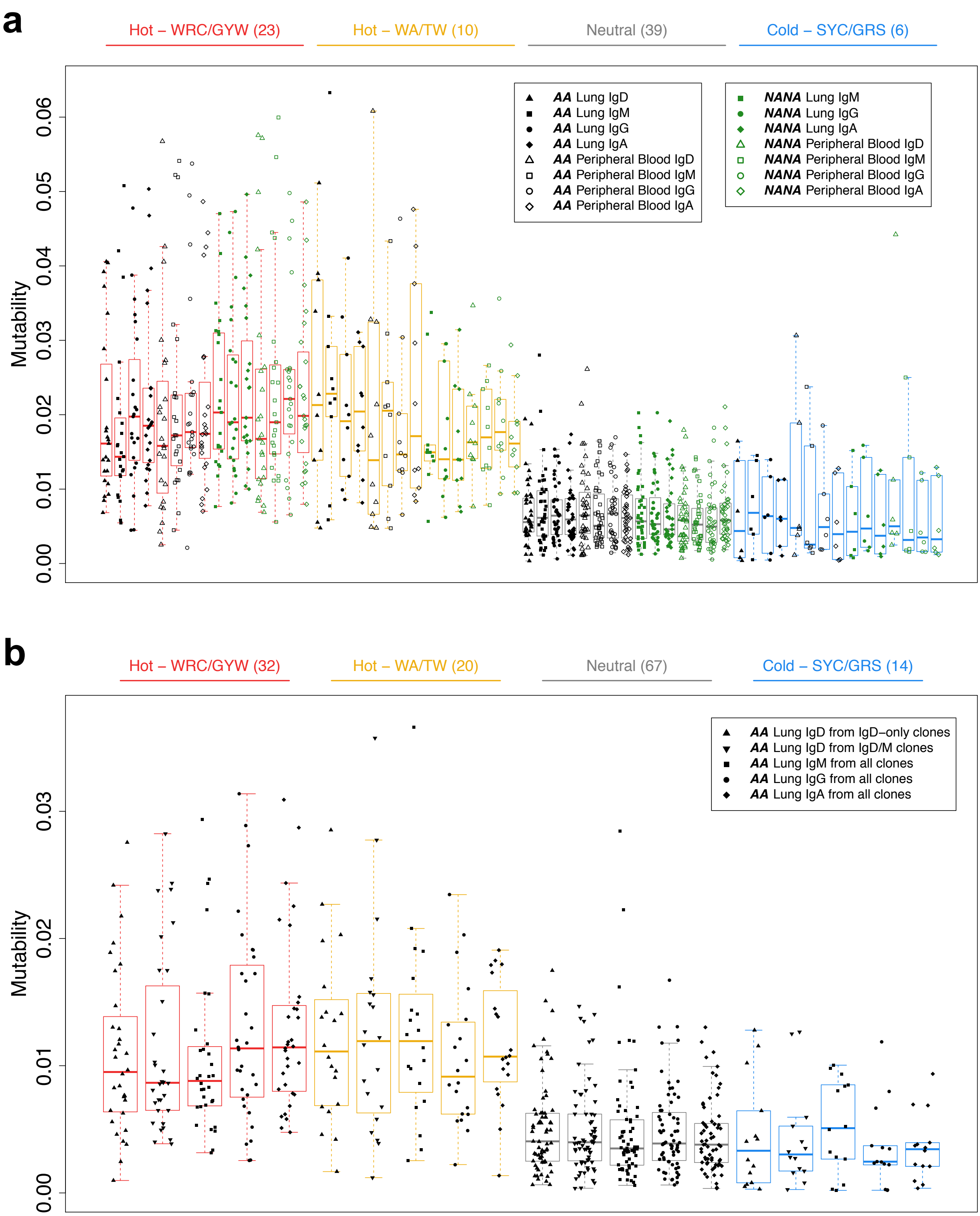

Supplementary Figure S2

Levels of somatic hypermutation (SMH) in hot- and cold spots suggest an AID initiated process. SMH targeting profiles were analyzed for (A) 78 5-mer motifs from each of the eight sample-isotype combinations from the asthmatic subject *AA* and the healthy subject *NANA* and (B) 133 5-mer motifs

from the *AA* bronchial mucosal sequences divided into IgD sequences from IgD-only clones and those from IgD/M clones, as well as IgM, IgG, and IgA sequences from all clones. IgD-only clones contain IgD but no IgM. IgD/M clones contain both IgD and IgM. The motifs were identified in the VH region up to and including the conserved cysteine at codon 104 according to the IMGT unique numbering scheme (SR4) using the synonymous, 5-mer, functional criteria (21). To reduce bias from clonal expansion, only the single most highly mutated sequence per isotype was analyzed from each clonal lineage. Mutability of a given 5-mer was directly measured using the "createMutabilityMatrix" function from SHazaM (13) (v0.1.7) and only motifs with at least 80 observed mutations in the central nucleotide were included. Within each sample-isotype combination, mutability was re-normalized such that mutability of the 5-mer motifs summed to one. IgE and *NANA* bronchial mucosal IgD were excluded from this analysis because an insufficient number of sequences was available. (A) 23 and (B) 32 of the 5-mers contained a WRC/GYW hotspot motif (red), (A) 10 and (B) 20 a WA/TW hotspot motif (yellow), (A) 39 and (B) 67 are neither hotspots nor coldspots (grey) and (A) 6 and (B) 14 are known SYC/GRS coldspots (blue). Each dot represents a 5-mer motif, and each box covers the 25th-75th percentiles of the mutability rates of the 5-mer motifs in its corresponding group, with the horizontal bar indicating the median. All of the analyzed known hotspots (red and yellow) show a relatively high level of mutability with medians around 0.02 but with some variability between sample types and isotypes. In contrast, the neutral and cold spots display much lower mutability and variability across sample types and isotypes. These patterns are consistent with SHM initiated by activation-induced cytidine deaminase (AID).

Supplementary Figure S3

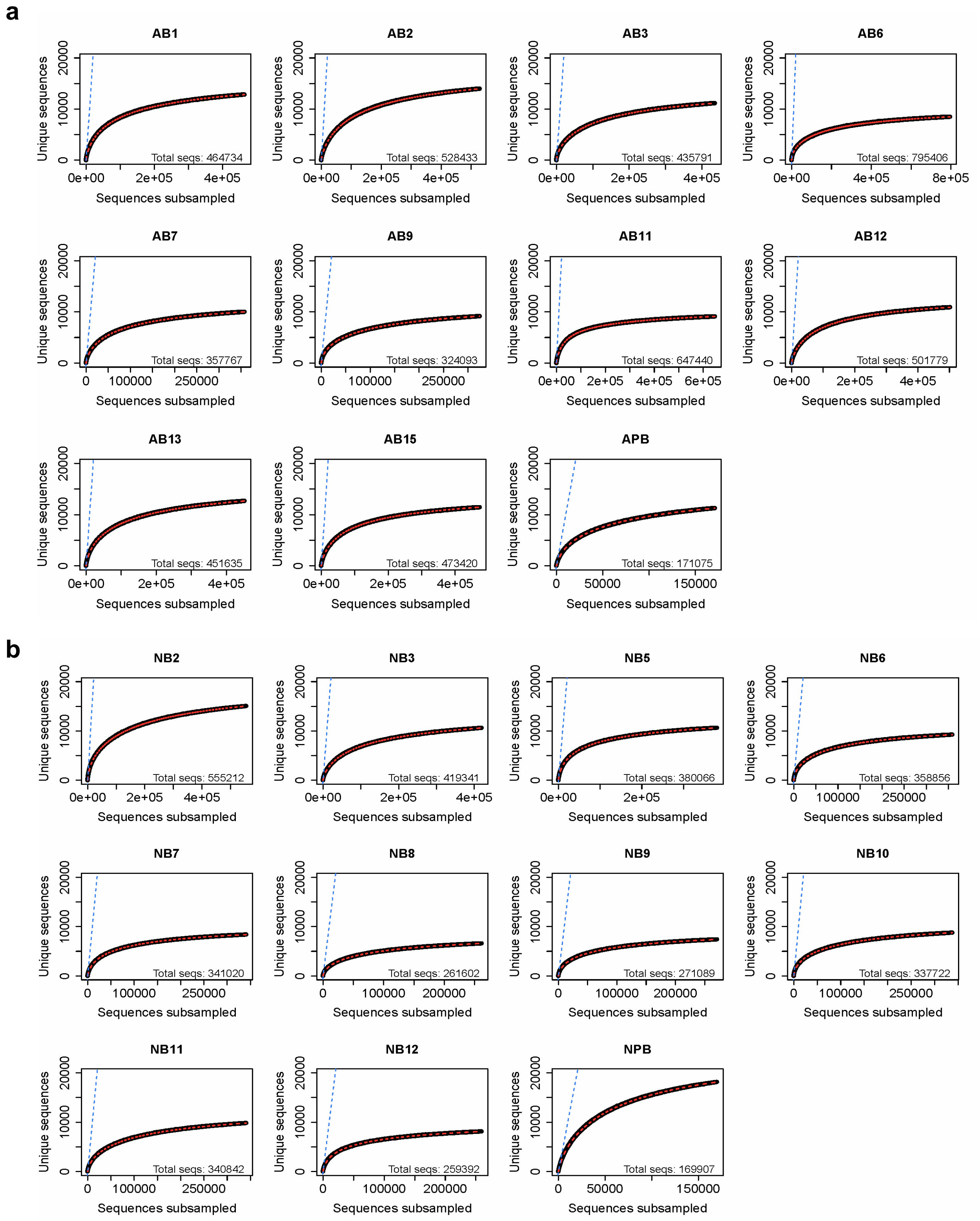

Supplementary Figure S3

All samples exhibited good sequencing coverage. Rarefaction analysis was carried out by calculating the number of unique sequences that result from randomly sampling a given number of sequences for

each sample from (A) the asthmatic subject *AA* and (B) the healthy subject *NANA*. Since sequences with fewer than five copies were filtered from the data to reduce sequencing errors, the copy number of each unique sequence was reduced by four prior to rarefaction analysis so that the minimum copy number was one. The flattening of the rarefaction curve in all biopsy samples and peripheral blood suggests that additional sequencing would identify few new immunoglobulin transcripts. The dashed blue line is the 1:1 ratio line. AB=asthmatic bronchial biopsy; APB=asthmatic subject peripheral blood; NB=non-atopic, non-asthmatic bronchial biopsy; NPB= non-atopic, non-asthmatic peripheral blood.

Supplementary References

SR1. Vander Heiden JA, Yaari G, Uduman M, Stern JN, O'Connor KC, Hafler DA, et al. pRESTO: a toolkit for processing high-throughput sequencing raw reads of lymphocyte receptor repertoires. *Bioinformatics* (2014) 30(13):1930-2. doi: 10.1093/bioinformatics/btu138.

SR2. Felsenstein J. PHYLIP - phylogeny inference package (version 3.2). *Cladistics* (1989) 5:164-6.

SR3. Csardi GT, Nepusz. The igraph software package for complex network research. *InterJournal Complex Systems* (2006) 1695.

SR4. Lefranc MP, Pommie C, Ruiz M, Giudicelli V, Foulquier E, Truong L, et al. IMGT unique numbering for immunoglobulin and T cell receptor variable domains and Ig superfamily V-like domains. *Dev Comp Immunol* (2003) 27(1):55-77.
